# Sex Chromosome Mosaicism and Hybrid Speciation among Tiger Swallowtail Butterflies

**DOI:** 10.1371/journal.pgen.1002274

**Published:** 2011-09-08

**Authors:** Krushnamegh Kunte, Cristina Shea, Matthew L. Aardema, J. Mark Scriber, Thomas E. Juenger, Lawrence E. Gilbert, Marcus R. Kronforst

**Affiliations:** 1 Section of Integrative Biology, University of Texas, Austin, Texas, United States of America; 2 FAS Center for Systems Biology, Harvard University, Cambridge, Massachusetts, United States of America; 3 Department of Ecology and Evolutionary Biology, Princeton University, Princeton, New Jersey, United States of America; 4 Department of Entomology, Michigan State University, East Lansing, Michigan, United States of America; Yale University, United States of America

## Abstract

Hybrid speciation, or the formation of a daughter species due to interbreeding between two parental species, is a potentially important means of diversification, because it generates new forms from existing variation. However, factors responsible for the origin and maintenance of hybrid species are largely unknown. Here we show that the North American butterfly *Papilio appalachiensis* is a hybrid species, with genomic admixture from *Papilio glaucus* and *Papilio canadensis*. *Papilio appalachiensis* has a mosaic phenotype, which is hypothesized to be the result of combining sex-linked traits from *P. glaucus* and *P. canadensis*. We show that *P. appalachiensis*' Z-linked genes associated with a cooler thermal habitat were inherited from *P. canadensis*, whereas its W-linked mimicry and mitochondrial DNA were inherited from *P. glaucus*. Furthermore, genome-wide AFLP markers showed nearly equal contributions from each parental species in the origin of *P. appalachiensis*, indicating that it formed from a burst of hybridization between the parental species, with little subsequent backcrossing. However, analyses of genetic differentiation, clustering, and polymorphism based on molecular data also showed that *P. appalachiensis* is genetically distinct from both parental species. Population genetic simulations revealed *P. appalachiensis* to be much younger than the parental species, with unidirectional gene flow from *P. glaucus* and *P. canadensis* into *P. appalachiensis*. Finally, phylogenetic analyses, combined with ancestral state reconstruction, showed that the two traits that define *P. appalachiensis*' mosaic phenotype, obligatory pupal diapause and mimicry, evolved uniquely in *P. canadensis* and *P. glaucus*, respectively, and were then recombined through hybridization to form *P. appalachiensis*. These results suggest that natural selection and sex-linked traits may have played an important role in the origin and maintenance of *P. appalachiensis* as a hybrid species. In particular, ecological barriers associated with a steep thermal cline appear to maintain the distinct, mosaic genome of *P. appalachiensis* despite contact and occasional hybridization with both parental species.

## Author Summary

Hybridization between two species may sometimes lead to the formation of a third species. While this phenomenon is of interest because it can generate new species from existing variation, it is rare in animals. Therefore, the factors responsible for the origin and maintenance of hybrid species are largely unknown. Here we show that the butterfly *Papilio appalachiensis* appears to be a hybrid species, which received nearly equal contributions to its genome from *P. glaucus* and *P. canadensis*. Two sets of traits define *P. appalachiensis*' hybrid phenotype: like *P. canadensis* it inhabits a cold habitat and has a single generation every year, and like *P. glaucus* it mimics a toxic butterfly and its females are dimorphic. The genes responsible for these traits are on two different sex chromosomes. Our genetic data show that *P. appalachiensis* inherited the sex chromosome associated with the cold habitat from *P. canadensis*, whereas it inherited the sex chromosome associated with mimicry and dimorphism from *P. glaucus*. Historical climate change may have facilitated the origin of *P. appalachiensis*, which our data suggest coincided with the last interglacial period in North America. This example shows the importance of sex-linked traits and a potential role for natural selection in the origin and maintenance of hybrid species.

## Introduction

Inter-specific hybridization is widespread in nature and may have important consequences in evolution, from the transfer of adaptive alleles between species to the formation of hybrid species [1]–[8]. Although prevalent in plants, hybrid speciation is apparently uncommon among animals, which may be explained by two factors: (a) hybrid populations may be weakly reproductively isolated from the parental species, so instead of maintaining their genomic identity, they may fuse with one of the parental species by backcrossing, and (b) hybrids may be unable to compete for ecological niches already occupied by parental species [3]–[5]. Hence, hybrid populations are likely to evolve as a species only when hybrids mate assortatively with other hybrids [7], and/or when they adapt to a new environment [6]. Both processes involve selection on specific phenotypic and ecological traits that favor the origin and maintenance of hybrid species. Selective introgression of genes responsible for divergent ecological traits from both parental species into a hybrid species would provide strong evidence for natural selection in the origin of a hybrid species [9]. However, selective regimes that may favor the formation of hybrid species are poorly understood and genetic evidence for selective introgression of ecologically important traits in a hybrid species is limited [9], [10]. For instance, several putative examples of animal hybrid species are known, ranging from fish to crustaceans and insects [11], but the potential role of natural selection in their origin and maintenance is largely unknown. Also, there are no examples of hybrid species that maintain their genomic identity while in contact with both parental species, which is important because sympatry may suggest that hybrid species are maintained by natural selection. Indeed, other known or suspected hybrid species are allopatric relative to either one or both parental species [6], [7], [11]. Here we show that the tiger swallowtail butterfly *Papilio appalachiensis* exhibits many hallmarks of a hybrid species, including a mosaic genome derived from *P. glaucus* and *P. canadensis*. At the same time, its genome is significantly differentiated while being in contact with both its parental species. We further show that its hybrid phenotype, which appears to be under selection, was produced by combining sex chromosomes of its parental species. Thus, *Papilio appalachiensis* may be an informative case for understanding the role of natural selection in both the origin and maintenance of hybrid species.

**Figure 1:**
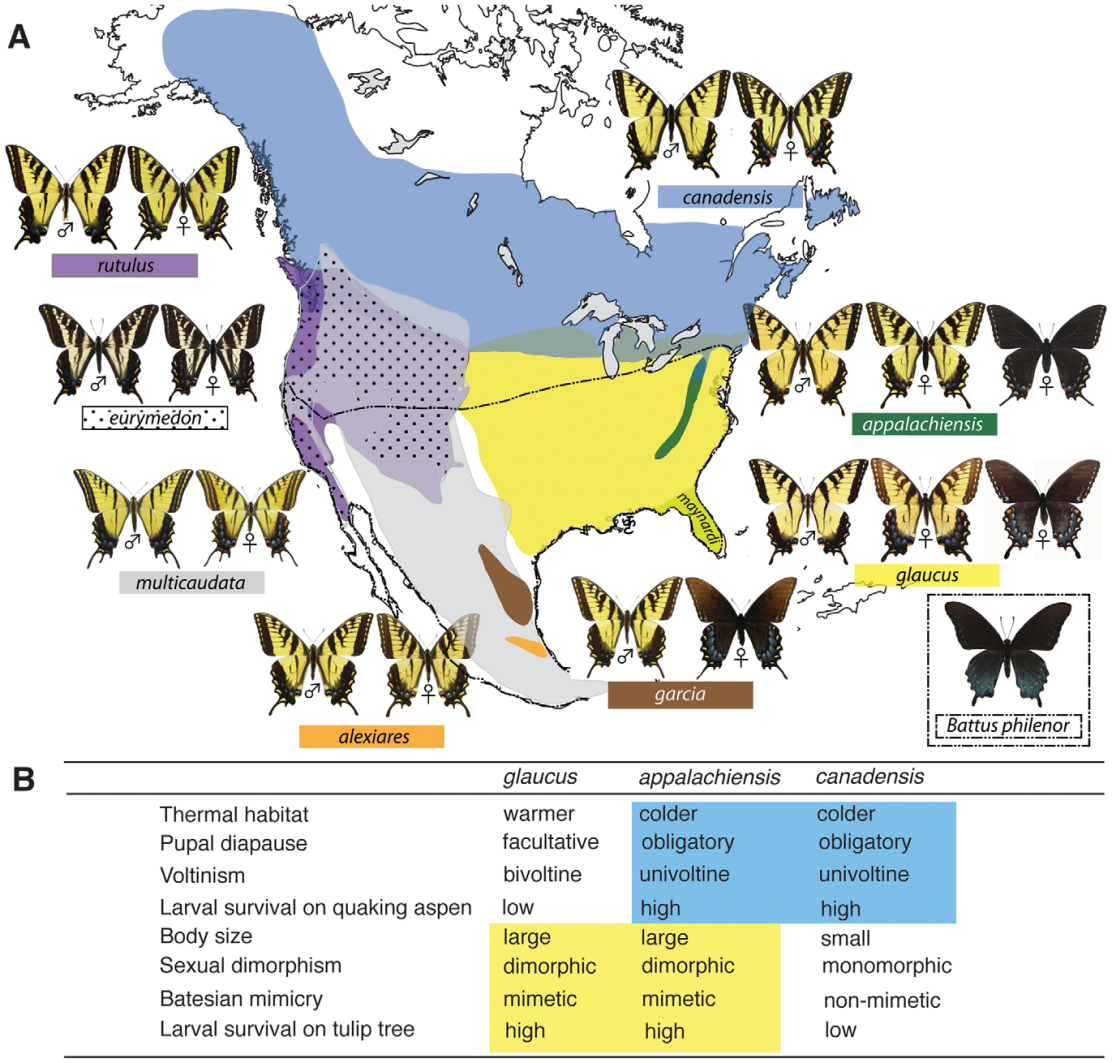
The distributional ranges and hybrid zones of tiger swallowtails, and the hybrid phenotype of *Papilio appalachiensis*. (A) *Papilio appalachiensis* is endemic to mid- and high elevations in the Appalachian Mountains and sympatric with *glaucus* throughout its range, but presumably parapatric with *canadensis* in its northernmost range [35], [36] (see Materials and Methods). Also shown is the range of *Battus philenor*, Batesian model for the mimetic *glaucus*, *appalachiensis* and *garcia* melanic female forms. (B) Ecological and morphological differentiation between *glaucus* and *canadensis*, and their admixture in *appalachiensis* [35]–[38] (also see Figure S1).

Tiger swallowtail butterflies of North America (Figure 1A) are a monophyletic group consisting of eight closely-related species. Sister species *glaucus* and *canadensis* show clear and strong evidence for interspecific divergence and multiple forms of reproductive isolation: one-way assortative mate preference [12], [13], reduced hatching success of hybrid eggs [13], [14], Haldane's Rule and hybrid incompatibility in *glaucus/canadensis* crosses [14], [15], divergent thermal habitat preference [16]–[18], differential host-plant preference/usage [18]–[22] and larval development times [23], and differential survival on preferred host plants [14], [19], [24] (also see Materials and Methods). This broad range of isolating mechanisms shows that *glaucus* and *canadensis* are good biological species [13], [25]. They have diverged ecologically along a steep thermal gradient in spite of hybridizing across a long and narrow hybrid zone [25]–[27]. This hybrid zone is characteristically bimodal, where hybrids are uncommon relative to the parental species [28] (also see Materials and Methods). The hybrid zone is maintained by thermal ecology: *glaucus* is adapted to a warmer thermal habitat compared to *canadensis*. This has shaped key ecological differences between the two species, including voltinism and pupal diapause (Figure 1B). These traits are Z-linked (Lepidoptera have ZZ males and ZW females) [15], [21], [29]. The thermal landscape also contributes indirectly to the evolution of Batesian mimicry, which is under frequency-dependent selection and produces sexual dimorphism and female dimorphism in *glaucus* [30], [31]. *Papilio glaucus* is palatable to vertebrate predators, and has two female forms: a yellow, male-like, non-mimetic form and a melanic form that mimics the chemically defended *Battus philenor* (Figure 1A) [32]. The distribution of *B. philenor* is limited in the north by its larval host plant, producing a latitudinal gradient in the frequency of the *glaucus* mimetic female form [30], [33], [34]. The Mendelian locus controlling female color in *glaucus* is W-linked, with a Z-linked mimicry enabler allele nearly fixed in *glaucus* and a mimicry suppressor allele fixed in *canadensis* [26], [29].

Recently described *P. appalachiensis* appears to be a phenotypic mixture of *glaucus* and *canadensis*, with which it is sympatric and parapatric, respectively (Figure 1A), but is apparently reproductively isolated from both (see Materials and Methods, and below). It shows affinity with *canadensis* for traits related to its cooler thermal habitat but with *glaucus* for traits related to mimicry and sexual/female dimorphism (Figure 1B) [35]–[38]. This mosaic phenotype, which is critical to its existence in cooler, high-elevation habitats along the Appalachian Mountains but within the range of *B. philenor*, has led to the suggestion that *appalachiensis* may be a hybrid species [35], [37]. In support of this hypothesis, preliminary allozyme data have shown that *appalachiensis* has a *canadensis*-like allele at the Z-linked *Lactate Dehydrogenase* (*Ldh*) gene and a *glaucus*-like allele at the Z-linked *Phosphogluconate Dehydrogenase* (*Pgd*) gene [38].

Here we test the hypothesis that *appalachiensis* is a hybrid species with genomic admixture from *glaucus* and *canadensis*. If it is a hybrid species, its specific mixture of traits combined with the known sex-linkage of these traits make a clear prediction regarding the ancestry of *appalachiensis*' sex chromosomes: *appalachiensis* has inherited most of its Z chromosome from *canadensis* and its W chromosome from *glaucus*. We also test whether it has genome-wide admixture from the parental species, as expected of hybrid species that are formed with little subsequent backcrossing with parental species. In addition, we test alternative hypotheses regarding the apparently mixed genotype and phenotype of *appalachiensis*. Finally, we estimate divergence times between *glaucus*, *canadensis* and *appalachiensis* and study the evolutionary history of the traits that make up *appalachiensis*' hybrid phenotype in order to shed light on the evolutionary dynamics of the origin and maintenance of this hybrid species.

## Results/Discussion

**Figure 2:**
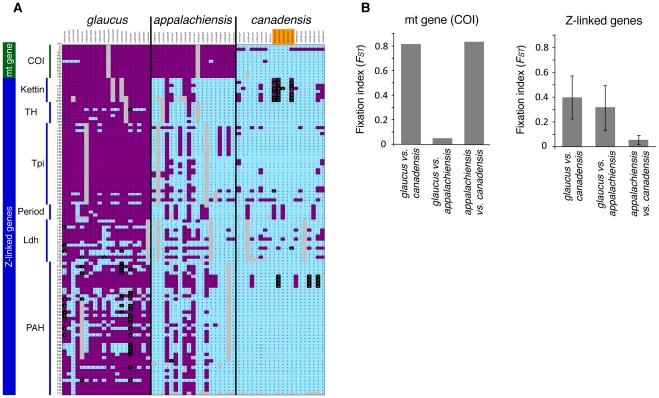
Genotypic differentiation between *glaucus* and *canadensis*, and the mismatch in mitochondrial and Z-linked genes in *appalachiensis*. (A) *appalachiensis* genotypes at loci that were significantly different (p<0.001) between *glaucus* and *canadensis*, as judged by *F_ST_* values from a locus-by-locus AMOVA comparing *glaucus* and *canadensis*. Genotypes are nucleotide bases at specific SNP or indel polymorphisms, which can be diploid (Z-linked polymorphisms scored in males) or haploid (mtDNA, and Z-linked polymorphisms scored in females). Color code: purple: genotypes characteristic of *glaucus*; light blue: genotypes characteristic of *canadensis*; black: heterozygotes; grey: missing data; orange: late flight *canadensis*. (B) Species pair-wise *F_ST_* values for the mitochondrial and Z-linked genes (see Table S4 for individual values for each gene and species pair-wise comparisons).

We tested the prediction that *appalachiensis* has mosaic sex chromosomes by sequencing six genes along the length of the Z chromosome and the mitochondrial *cytochrome oxidase I* (*COI*) gene (Materials and Methods, Table S2). While there are currently no known W-linked molecular markers in butterflies, we can infer the history of the W chromosome by sequencing mitochondrial DNA because both are maternally inherited and hence linked in Lepidoptera (see Materials and Methods). As predicted, our sequence data showed clear genetic discordance with *appalachiensis* having a mitochondrial genome similar to that of *glaucus*, and a Z chromosome similar to *canadensis* (Figure 2, Tables S3 and S4).

**Figure 3:**
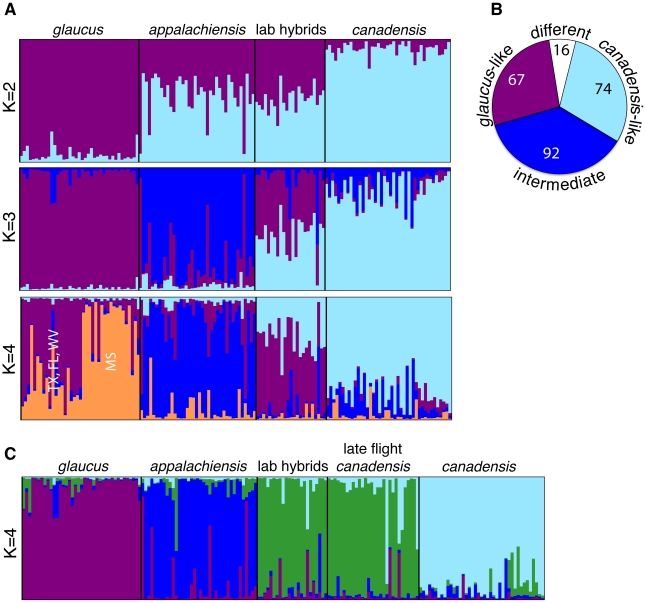
Genomic admixture in *appalachiensis* showing its hybrid origin and its contrast with laboratory-generated hybrids and late flight *canadensis*. (A) Population clustering of AFLP data in STRUCTURE under the assumption of two, three and four populations, comparing *appalachiensis* with laboratory-generated *glaucus* x *canadensis* hybrids. (B) *appalachiensis* AFLP allele frequencies with respect to *glaucus* and *canadensis*, based on species pair-wise locus-by-locus AMOVAs. Allele frequencies of “*glaucus*-like” AFLPs were significantly different from *canadensis*, “*canadensis*-like” AFLPs were significantly different from *glaucus*, “intermediate” were intermediate between *glaucus* and *canadensis* but significantly different from neither, and “different” were significantly different from both *glaucus* and *canadensis*. (C) Population clustering in STRUCTURE under the assumption of four populations, showing genomic similarity between the laboratory-generated hybrids and late flight *canadensis*, and distinctiveness of *appalachiensis* (also see Figure S2). For (A) and (C), admixture proportions of the sampled individuals, rather than their assignment probabilities, are shown.

While the observed sex chromosome mosaicism suggests that *appalachiensis* evolved by combining key, sex-linked ecological traits from *glaucus* and *canadensis*, it does not tell us whether *appalachiensis* has a hybrid ancestry across the rest of its genome. Such widespread genomic admixture would be expected for a hybrid species formed with little subsequent backcrossing with the parental species. Alternatively, *appalachiensis* may be largely similar to one parental species across its genome, with a selectively introgressed W (and mtDNA) or Z chromosome from the other parental species [36]. To test these alternatives, we screened 2,035 nuclear amplified fragment length polymorphism (AFLP) markers and identified 249 that distinguished *glaucus* from *canadensis*. We then genotyped these ancestry informative markers in *glaucus*, *canadensis*, *appalachiensis* and laboratory-generated *glaucus* x *canadensis* F1 and F2 hybrids. We used the program STRUCTURE to genetically cluster the samples under the assumption that they represented two, three or four admixed populations. This analysis revealed that *appalachiensis* has genome-wide admixture indicative of historical hybridization between *glaucus* and *canadensis* (Figure 3A, Figure S2). Under the assumption of two populations (K = 2), *appalachiensis* and laboratory-generated hybrids showed similar and nearly equal admixture from *glaucus* and *canadensis*. At K = 3, *appalachiensis* formed its own cluster, and at K = 4 it remained a distinct cluster while subdivision within *glaucus* became apparent. In contrast, at K = 3 and K = 4, the laboratory-generated hybrids continued to show similar admixture from both parental species. A locus-by-locus analysis of molecular variance (AMOVA) of the AFLP dataset further confirmed widespread genomic admixture in *appalachiensis*: allele frequencies at 67 AFLP markers were similar to *glaucus* and significantly different from those of *canadensis*, 74 AFLP markers were similar to *canadensis* and significantly different from *glaucus*, and 92 markers had frequencies intermediate between *glaucus* and *canadensis* but significantly different from neither (Figure 3B). This analysis also revealed 16 AFLPs for which the frequency in *appalachiensis* was significantly different from both parental species (Figure 3B). Importantly, co-occurring *appalachiensis* and *glaucus* individuals, as identified morphologically at the time of collection from Spruce Knob in May and June 2006 (see Materials and Methods), showed species-specific sequences (Figure 2A) and AFLP signatures (Figure 3A). This suggests that these two co-occurring species are genetically distinct and that the clusters seen in Figure 2 and 3 are not merely geographically isolated subpopulations of the same species.

**Figure 4:**
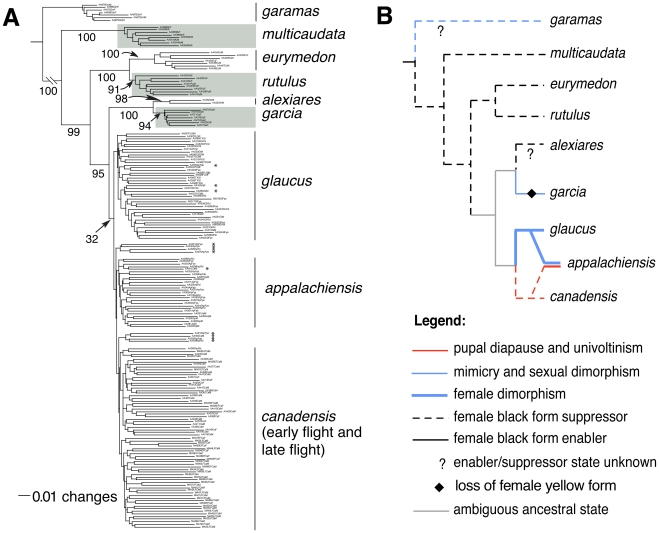
Phylogenetic relationships and character evolution among tiger swallowtails. (A) AFLP-based neighbor-joining tree, with percentage bootstrap support shown for branches. The ten *appalachiensis* and two *canadensis* samples that cluster outside their species are marked with asterisks. (B) Character evolution based on the AFLP phylogeny.

Examining the evolutionary context of the hybrid phenotype of *appalachiensis* requires a well-resolved phylogeny of tiger swallowtails. However, resolving this phylogeny from sequence data is challenging owing to recent divergence, incomplete lineage sorting and ongoing hybridization [39], [40] (also see Materials and Methods). Therefore, we used 1,607 polymorphic AFLP markers to generate a bootstrap-supported neighbor-joining tree of the entire clade. All tiger swallowtail nodes were strongly supported, except the *glaucus*, *canadensis* and *appalachiensis* clades (Figure 4A; also see Table S5). Within the *glaucus-canadensis-appalachiensis* groups, individuals formed species-specific clusters with only 12 out of the 184 samples clustering outside their own species (Figure 4A). We resolved the ancestral nodes between *appalachiensis, glaucus* and *canadensis* based on Figure 2 and Figure 3, and mapped relevant ecological traits (Table S6) on the resulting phylogeny (Figure 4B). We found that the mimetic female form suppressor is basal and widespread among tiger swallowtails. Mimicry and the enabler either evolved independently in *garcia* and *glaucus*, or were lost in *alexiares* and *canadensis*. The ancestral yellow female form was uniquely lost in *garcia*, whereas female dimorphism was uniquely gained in *glaucus* and then introgressed into *appalachiensis*. Similarly, obligatory pupal diapause and univoltinism evolved uniquely in *canadensis* and introgressed into *appalachiensis*. Thus, we not only identified the specific traits that recombined to form the hybrid species, we also traced the origin and spread of those traits through evolutionary history, down to the time they were brought together through hybridization to generate a new species.

The evolutionary history and direction of introgression in *appalachiensis* contrasts with the phenomenon of *canadensis* “late flight”, which has emerged recently near the northern limit of the *glaucus-canadensis* hybrid zone in Vermont [38], [41]. The late flight occurs in July and is allochronic relative to true (“early flight”) *canadensis*, which flies in May and June [38], [41]. The late flight has been hypothesized to be a result of hybridization between *glaucus* and *canadensis*, potentially representing an early stage in the evolution of an *appalachiensis*-like entity [41]. We used our DNA sequence and AFLP data to test whether late flight *canadensis* is of hybrid origin, and whether it is similar to *appalachiensis*. We found that unlike *appalachiensis*, the late flight is entirely *canadensis*-like at both its mitochondrial and Z-linked genes, except for three late flight individuals that were heterozygous for *glaucus*- and *canadensis*-like haplotypes at *Kettin* (Figure 2A). However, AFLP data show a strong signature of genome-wide admixture in late flight individuals (Figure 3C). Furthermore, the AFLP signature of the late flight is indistinguishable from that of laboratory-generated *glaucus* x *canadensis* F1 and F2 hybrids but distinct from *appalachiensis* (Figure 3C, Figure S2B), as expected if the late flight is a result of recent hybridization. The recent history of hybridization in the late flight is also supported by coalescent simulations (Figure S4, also see below and in Materials and Methods). Together, these data show that its history of introgression is dissimilar to *appalachiensis*. The late flight has the potential to speciate through allochronic flight period and larval host plant specialization [20], [41]; however, unlike *appalachiensis*, it may be a transient phase in the northward movement of the *glaucus-canadensis* hybrid zone in a changing thermal landscape. Moreover, although selection may maintain some characteristics of a mosaic sex chromosome in the late flight [20], [37], our data suggest that the late flight may not be experiencing natural selection for the same sex chromosome mosaicism that seems to have been essential in generating and maintaining the hybrid phenotype of *appalachiensis*.

**Figure 5:**
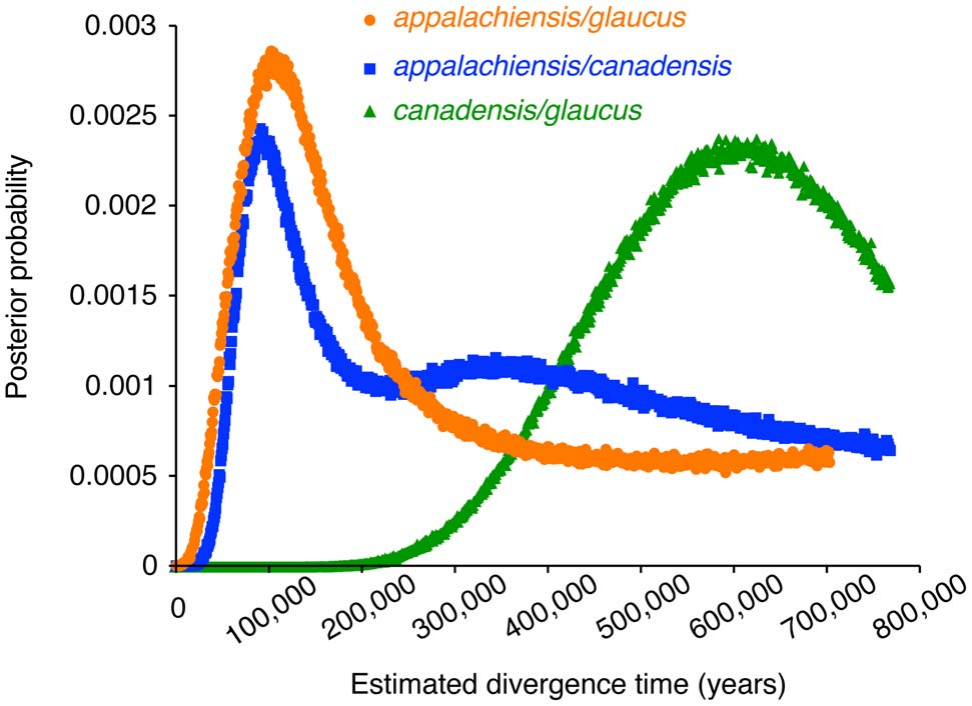
Estimated divergence times between the parental *glaucus* and *canadensis* and the hybrid *appalachiensis*. Dates of divergence estimated by IMa2 are: (a) *appalachiensis* and *glaucus*: ca 100,000 years ago, (b) *appalachiensis* and *canadensis*: ca 90,000 years ago, (c) *glaucus* and *canadensis*: ca 580,000 years ago.

**Figure 6:**
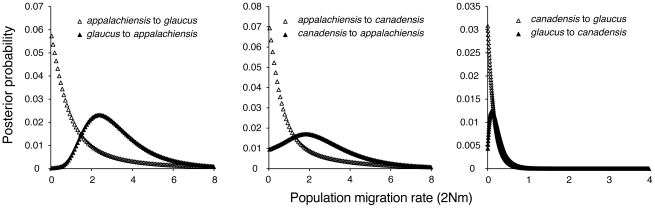
Estimated gene flow among *appalachiensis*, *glaucus*, and *canadensis*. Gene flow was estimated as the population migration rate or 2Nm, which is equivalent to the historical average number of immigrants between species per generation: *glaucus* to *appalachiensis*: 2.3; *canadensis* to *appalachiensis*: 1.8; *appalachiensis* to either *glaucus* or *canadensis*: 0; *glaucus* to *canadensis*: 0.1; *canadensis* to *glaucus*: 0.

We tested two alternative hypotheses for the apparent hybrid ancestry of *appalachiensis*: (1) *appalachiensis*, *glaucus* and *canadensis* diverged from one another in a standard bifurcating manner and ancestral variation sorted out in such a way that *appalachiensis* now appears to have a hybrid genotype and phenotype, and (2) *appalachiensis* is not a reproductively isolated species but a very recent hybrid population that is constantly supplied by ongoing hybridization between *glaucus* and *canadensis*. We addressed the first hypothesis with our DNA sequence data by estimating divergence times and rates of historical introgression among the three species with the coalescent approach implemented in IMa2. Instead of supporting a bifurcating model of speciation, this analysis showed that *glaucus* and *canadensis* diverged approximately 580,000 years ago, whereas *appalachiensis* diverged from both the parental species approximately 100,000 years ago during the Pleistocene (Figure 5). The IMa2 analysis also showed essentially unidirectional introgression from *glaucus* and *canadensis* into *appalachiensis* (Figure 6), consistent with the hypothesized hybrid origin of *appalachiensis*.

We tested the second hypothesis by comparing among the three focal species: (a) the extent of linkage-disequilibrium, and (b) the proportion of species-specific genetic polymorphisms and haplotypes. Recent hybridization is expected to produce elevated linkage disequilibrium as a result of bringing together two distinct chromosomal copies from each parent species, the linkage disequilibrium decaying through successive generations as a result of recombination [42]. Indeed, linkage disequilibrium in both our DNA sequence and AFLP data was elevated in the laboratory-generated hybrids and late flight *canadensis*, both of which are products of very recent hybridization (Table 1). In contrast, linkage disequilibrium was significantly lower in *appalachiensis* and within the range of that seen in *glaucus* and *canadensis* (Table 1). Together, this evidence supports *appalachiensis*' historical origin and highlights the differences in the hybrid history between *appalachiensis*, late flight *canadensis* and laboratory-generated hybrids. If *appalachiensis* is indeed an old and subsequently isolated hybrid species, then it should possess unique mutations that it would have accumulated since it split from its parental species. These unique mutations should be detectable even if their number may be relatively small and they may not be fixed given: (a) the short estimated divergence time of ca 100,000 years, and (b) possibly low level of ongoing hybridization with the parental species. We calculated the proportion of species-specific single nucleotide polymorphisms and haplotypes among the three focal species based on our sequence data from seven genes (Table 2). The number and proportion of species-specific polymorphisms among *glaucus*, *canadensis* and *appalachiensis* varied greatly among genes and species, but all three species had a substantial number of species-specific polymorphisms (Table 2). The proportion of species-specific polymorphisms averaged across the seven genes was 0.58 (±0.221) for *canadensis*, 0.59 (±0.158) for *glaucus*, and 0.41 (±0.19) for *appalachiensis*. Thus, *appalachiensis* had lower average species-specific polymorphisms compared to its parental species, as expected from its younger age, but the difference was not significant (ANOVA, *F*_(2,18)_ = 1.90, *p* = 0.179). We conclude that *appalachiensis* appears to have been isolated from the parental species long enough to accumulate species-specific mutations whose numbers are comparable with those of the parental species. This pattern is robust and holds true if we consider the number of species-specific haplotypes instead of polymorphisms: the proportion of species-specific haplotypes, averaged across all seven genes, was 0.71(±0.270) for *canadensis*, 0.9(±0.06) for *glaucus*, and 0.68(±0.098) for *appalachiensis* (ANOVA: *F*_(2,18)_ = 3.47, *p* = 0.0533) (Table 2). These polymorphism and haplotype data support the hypothesis that *appalachiensis* is a good species rather than a recent or constantly supplied hybrid population. Moreover, additional lines of evidence are in conflict with the second hypothesis: (a) *appalachiensis* has a unique morphological and behavioral phenotype (Figure 1B and Figure S1), (b) there is significant genetic differentiation among *appalachiensis*, *glaucus* and *canadensis*, and *appalachiensis* forms a very well-supported and distinct genomic cluster that is apparent in both the DNA sequence (Figure 2) and AFLP data (Figure 3, Table S5), (c) IMa2 simulations showed *appalachiensis* to have diverged from the parental species long ago (Figure 5), and (d) *Papilio appalachiensis* is parapatric with *canadensis* and occurs southward to Georgia in the southern Appalachian Mountains, very far outside *canadensis*' range (Figure 1A; Materials and Methods). Therefore, *appalachiensis* could not be a product of ongoing hybridization between *glaucus* and *canadensis*. It is also important to note that its phenotype is unlike that of the laboratory-generated *glaucus* x *canadensis* hybrids.

**Table 1:** Estimates of linkage disequilibrium, arranged from highest to lowest values, with mean ± SD.

AFLPs	Z-linked genes
laboratory-generated hybrids = 3.50±1.93late-flight *canadensis* = 3.22±1.65*canadensis* = 2.56±0.98*appalachiensis* = 2.35±1.02*glaucus* = 2.23±1.52	*canadensis* = 21.57±18.29*appalachiensis* = 19.92±12.48*glaucus* = 9.12±4.98

Linkage disequilibrium was calculated as pair-wise associations among all polymorphisms, and summarized below as the average percentage of polymorphisms that were significantly (p≤0.01) associated with each other. For AFLPs, differences in levels of linkage disequilibrium among all the groups were highly significant (p<0.0001), except among *appalachiensis* and *glaucus* (p = 0.053). For Z-linked genes, *canadensis* and *appalachiensis* were not significantly different from each other (p = 0.214) but they are both different from *glaucus* (p<0.0001).

**Table 2:** Distribution of single nucleotide polymorphisms and haplotype diversity among *glaucus*, *canadensis*, and *appalachiensis*.

	Number of samples sequenced (number of sequences)	Number of single nucleotide polymorphisms in a species	Number (and %) of species-specific single nucleotide polymorphisms	Number of haplotypes in a species	Number (and %) of species-specific haplotypes
***COI***					
* canadensis*	24	11	7 (64%)	11	10 (91%)
* glaucus*	56	18	10 (56%)	19	15 (79%)
* appalachiensis*	28	8	3 (37%)	7	4 (57%)
***KET***					
* canadensis*	20 (22)	37	25 (68%)	21	17 (81%)
* glaucus*	18 (18)	20	18 (90%)	15	13 (87%)
* appalachiensis*	17 (19)	30	15 (50%)	11	8 (73%)
***TH***					
* canadensis*	21 (21)	6	4 (67%)	8	2 (25%)
* glaucus*	34 (34)	24	14 (58%)	27	25 (93%)
* appalachiensis*	35 (35)	23	8 (35%)	19	12 (63%)
***TPI***					
* canadensis*	25 (27)	57	53 (93%)	20	17 (85%)
* glaucus*	30 (30)	19	9 (47%)	22	20 (91%)
* appalachiensis*	16 (17)	21	9 (43%)	10	7 (70%)
***PER***					
* canadensis*	25 (25)	8	2 (25%)	10	4 (40%)
* glaucus*	32 (32)	5	3 (60%)	30	27 (90%)
* appalachiensis*	19 (19)	21	16 (76%)	9	5 (56%)
***LDH***					
* canadensis*	26 (26)	46	19 (41%)	26	23 (88%)
* glaucus*	28 (28)	38	23 (61%)	28	27 (96%)
* appalachiensis*	16 (16)	49	15 (31%)	14	11 (79%)
***PAH***					
* canadensis*	20 (27)	46	21 (46%)	26	23 (88%)
* glaucus*	20 (24)	109	43 (39%)	24	23 (96%)
* appalachiensis*	18 (19)	100	14 (14%)	15	12 (80%)

For some Z-linked genes, the number of sequences differed from the number of samples sequenced because of heterozygosity in males.

### Conclusions

Our data show that *Papilio appalachiensis* displays multiple hallmarks of a hybrid species. Furthermore, it has two unique features that inform us on the dynamics of hybrid speciation. First, it may potentially be the sole example of a hybrid species that spatially overlaps with both parental species. Second, its sex-linked ecologically important traits, and therefore sex chromosome mosaicism, appear to be under selection. In this case the sharp ecological clines along the latitudinal and altitudinal thermal gradients seem to maintain three, rather than two, spatially overlapping tiger swallowtail species. The evolution and persistence of *appalachiensis* in contact with its parental species suggests that hybridization among animals may result in selectively favored hybrid species that contribute to biodiversity. Moreover, sex chromosomes play an important role in speciation [43], and this has specifically been demonstrated in the Lepidoptera [44], [45], but this is the first example in which sex-linked traits seem to have contributed to hybrid speciation.

As a whole, our data suggest a scenario for the origin and maintenance of *appalachiensis*. During one of the late Pleistocene glacial retreats, *canadensis* populations retreated from their southern range while *glaucus* populations advanced northward and upward into the mountains. The changing thermal landscape likely brought the advancing *glaucus* populations into contact with a relict *canadensis* population in the Appalachian Mountains. The ensuing hybridization seems to have been largely unidirectional, with *canadensis* males preferentially mating with *glaucus* females, as they do today [12]. Hence, *appalachiensis* is now fixed for the *glaucus* mitochondrial genome, along with its W-linked female-limited mimicry and dimorphism. Both these traits are under frequency-dependent selection in the present *appalachiensis* habitat because with glacial retreats, selection for mimicry has also moved northward along with the range of *B. philenor*. However, the cold Appalachian thermal habitat ensured that the Z-linked genes associated with this lifestyle persisted from its *canadensis* ancestry in the proto-*appalachiensis* populations [16], [37]. This selection also persists today [38]. This unique combination of traits under varied selection may have been critical in helping *appalachiensis* evolve as a distinct species. The maintenance of genome-wide admixture in *appalachiensis* (Figure 3) also suggests that it was formed via a relatively brief burst of hybridization between the parental species, with little backcrossing with *glaucus* despite their continued sympatry throughout *appalachiensis*' range. A few other hybrid species have likely emerged in a similarly short span of time with potentially little backcrossing [8], [46], [47]. Interestingly, our estimated divergence times of approximately 100,000 and 90,000 years between *glaucus/canadensis* and *appalachiensis* (see Figure 5) fall precisely in the last interglacial period in North America, known as the Sangamonian Stage (125,000 to 75,000 years ago), which is congruent with the scenario just outlined. It may be possible to explore this scenario with additional population genetic data for these species.

*Papilio appalachiensis* may provide a rare genetic example of the creative role of hybridization in evolution [8]. Hybrids are often considered maladapted and viewed as lying in fitness valleys between the adaptive peaks that the parental species occupy in the adaptive landscape [3]. In case of tiger swallowtails, however, the northward and upward movement of the toxic *B. philenor* in the Appalachian Mountains may have created a new, unoccupied adaptive peak with selection for a combination of cold thermal habitat and mimicry. Neither of the parental species appears to be able to occupy this peak. Our results suggest an intriguing scenario in which the hybrid species (*appalachiensis*), with its precise combination of phenotypic traits, may have landed directly on this novel, unoccupied adaptive peak.

The primary goals of this work were to test whether *appalachiensis* exhibited genetic evidence of hybrid ancestry, mosaic sex chromosomes, and genome-wide admixture. Our results reveal several intriguing patterns that appear to support *appalachiensis*' status as a hybrid species and suggest an evolutionary scenario for its origin and maintenance. Our work also highlights specific areas where additional data will enrich our understanding of this system in particular and the dynamics of hybrid speciation in general. One such area is identifying the specific mechanisms that generate reproductive isolation between *appalachiensis* and both of its parental species. Our genetic data, and the low frequency of *glaucus/appalachiensis* hybrids from areas where they co-occur in West Virginia, suggest that these two species exhibit some reproductive isolation. However, the ecological, behavioral and genetic factors that contribute to this isolation remain unknown, as does the exact degree of isolation. Specifically, we do not currently understand the mechanisms of isolation or the precise geographic ranges and areas of overlap between *appalachiensis* and *canadensis*. In addition, more widespread sampling of *appalachiensis* from across its range would allow us to test its genomic integrity throughout the range, and also to estimate the extent to which it hybridizes with each of the parental species. Given the latitudinal range of *appalachiensis* relative to *glaucus* and *canadensis*, it is possible that *appalachiensis* experiences variable amounts of recent introgression with each of the two parental species. Our current sampling was limited so we were unable to test this hypothesis. However, genetic data for our *appalachiensis* sample from its southern range in North Carolina and co-occurring samples of *appalachiensis* and *glaucus* from West Virginia suggest that *appalachiensis* may be fairly homogeneous throughout its range, but more widespread sampling is warranted. Scriber [48] has recently pointed out that ongoing climate warming may diminish the cool thermal mountain refuges in the southern Appalachian Mountains of northern Georgia, western North Carolina and eastern Tennessee in *appalachiensis*' southernmost range. The changing thermal landscape may induce increased introgression of *glaucus* genes into *appalachiensis*, which may diminish the genomic contribution from *canadensis* in these *appalachiensis* populations. Clearly, the rich biological detail of this system holds promise to test various aspects of hybrid speciation and persistence in animals.

## Materials and Methods

### Study System

#### The parental species, *P. glaucus* and *P. Canadensis*

Tiger swallowtails (*Papilio glaucus* species group) form a small, monophyletic species group of the American subgenus *Pterourus* of *Papilio* [40]. Many tiger swallowtail species look similar in general appearance, although they have consistent differences in ecological traits as well as larval, pupal and adult morphology [49], [50]. Nonetheless, there had been much confusion about subspecies and species status of three parapatric taxa: *rutulus*, *canadensis* and *glaucus*, since they occasionally hybridize when in contact at their range margins [50] (also see Figure 1A). Due to the lack of complete sympatry and occasional hybridization, many earlier authors had treated *rutulus* and *canadensis* as subspecies of *glaucus* [25], [50], [51]. The three taxa have now been solidly established as distinct species through the past three decades of work by JMS and his collaborators. This work has revealed multiple mechanisms of reproductive isolation separating *glaucus* and *canadensis* that indicate that they are good biological species [12]–[25] (also see the Introduction).

Interspecific hybridization is common in nature and there are many well-studied interspecific hybrid zones [3], [4], [52], [53]. Several features have been used to distinguish interspecific hybrid zones from contact zones between subspecies, most importantly: (a) interspecific hybrid zones are marked by a reduction in hybrids relative to the parental forms (“bimodal hybrid zone”) [54], and (b) the maintenance of “genotypic clusters” in spite of hybridization [55]. The *glaucus/canadensis* hybrid zone meets these two hallmarks of interspecific hybrid zones: (a) hybrids make up less than 20% of the community in the very middle of the *glaucus/canadensis* hybrid zone [28], and (b) our present genetic data as well as previous allozyme work [29], [56], [57] show that *glaucus* and *canadensis* form distinct genotypic clusters despite hybridization. The rate of hybridization reported for *glaucus* and *canadensis* in the middle of the hybrid zone is within the range of rate of hybridization known for other well-established species pairs [52].

Lastly, more recent phylogenetic work has shown *rutulus* and *canadensis* to be well-separated from *glaucus* [40]. Our present work (Figure 2, Figure 3, Figure 4, Figure 5, Table S5) further reinforces the view that *rutulus*, *canadensis* and *glaucus* are genetically strongly diverged parapatric species, not subspecies, despite occasional hybridization.

#### The putative hybrid species, *P. appalachiensis*

*Papilio appalachiensis* has a distinct and readily recognizable phenotype spanning morphology, ecology and behavior, and this phenotype is maintained in sympatry/parapatry with the parental species, *glaucus* and *canadensis* [35], [36]. There are several factors that suggest that *appalachiensis* is a distinct species rather than a recent hybrid population. For example, its melanic female form is unlike any other tiger swallowtail species, and both the male and female wing patterns are unlike any laboratory-generated *F1* and *F2 glaucus* x *canadensis* hybrids. Similar to the *glaucus/canadensis* hybrid zone (see above), *appalachiensis* shows two hallmarks of a species: (a) it has a bimodal hybrid zone with *glaucus*, and (b) it forms a unique genetic cluster distinct from its parental species (see Figure 2 and Figure 3, and Results/Discussion). *Papilio appalachiensis* shows highly reduced rate of hybridization with *glaucus*, with which it completely overlaps in distribution (Figure 1A). Although *appalachiensis* is found at mid- and high elevations in the Appalachian Mountains and *glaucus* is usually found at lower elevations, our work shows that they co-occur widely both along the elevational and latitudinal gradients at least for several weeks during late May and June. For example, KK found fresh individuals of both species feeding side by side on the same flowering honeysuckle bushes below Spruce Knob, West Virginia, in May and June 2006. Thus, there is ample opportunity for hybridization between the two taxa. Nonetheless, we collected only 16 suspected *glaucus/appalachiensis* hybrids (phenotypically intermediate between *glaucus* and *appalachiensis*), along with 114 phenotypically pure *appalachiensis* and 172 phenotypically pure *glaucus* within the overlap zone of *appalachiensis* and *glaucus* from Tennessee to northeastern West Virginia. Thus, the proportion of suspected hybrids between the two species was very small, approximately 5.6%, making this a clearly bimodal hybrid zone. These numbers are well below the estimates of hybridization for many other hybridizing species [4] and point to reduced hybridization as one would expect for distinct species. As a clarification, individuals were scored as putative hybrids between *appalachiensis* and *glaucus* based on the diagnostic wing color patterns given in the original species description [35], [36], mainly: (1) square versus crescent-shaped submarginal yellow spots on the hindwing, (2) contiguous yellow band versus row of spots on forewing underside, and (3) extent of blue on the upper hindwing of females. They were first identified as probable hybrids by KK and then all specimens were blindly scored by H. Pavulaan, who independently arrived at the same conclusion (Pavulaan originally described *appalachiensis*). If our species assignments had been wrong, underestimating the true rate of hybridization based on phenotypic characters, the genetic clustering shown in Figure 2 and Figure 3 would have been very different.

The contact zone and nature of isolation between *appalachiensis* and *canadensis* are currently not well understood. These two species are believed to be sympatric in the Appalachian Mountains from West Virginia northward [35], [58]. Almost all of our *appalachiensis* samples were collected near Spruce Knob, West Virginia (Table S1, Figure S5), very near localities where *canadensis* reportedly occurs. Based on our published [37], [38] as well as unpublished work, we suspect that *canadensis* has recently moved northward along the Appalachian Mountains, perhaps completely out of West Virginia, due to ongoing climate warming. It is likely that *appalachiensis* and *canadensis* maintain a hybrid zone similar to the *glaucus-canadensis* hybrid zone, and this hybrid zone may also have moved northward due to the ongoing warming in this area. Current work by MLA and JMS aims to delineate the present northern boundary of *appalachiensis* distribution, the southern boundary of *canadensis* in the Appalachian Mountains, and the boundaries of the contact zone between the two species. However, based on current understanding [35], [58], *appalachiensis* and *canadensis* are narrowly sympatric or parapatric.

### Specimen Collection and Lab Methods

Table S1 lists details of the 244 wild-caught individuals and 23 laboratory-generated hybrids used for genetic analysis, and Figure S5 shows localities where these specimens were collected. We preserved bodies in ethanol and stored wings in glassine envelopes. We measured wingspan from the preserved wings using vernier calipers, from the base of the forewing to its tip (Figure S1). We extracted genomic DNA from legs and thoracic muscle tissue using DNeasy Blood & Tissue Kit (Qiagen, Germantown, MD).

We sequenced one mitochondrial (*Cytochrome oxidase I, COI*) and six Z-linked genes (*Kettin* (*Ket*), *Tyrosine hydroxylase* (*TH*), *Triosephosphate isomerase* (*Tpi*), *Period* (*Per*), *Lactate dehydrogenase* (*Ldh*) and *Phenylalanine hydroxylase* (*PAH*)) to study potential admixture in *appalachiensis* (NCBI accession numbers JF764373-JF764558, JF951433-JF951722). Primer sequences and lengths of aligned sequences are given in Table S2. PCR protocols used for previously published primers are given in the literature [27], [59]. We designed primers for *TH* and *PAH* by aligning sequences from *Bombyx mori* and *Papilio xuthus* (Table S2). Previous allozyme work had shown that *appalachiensis* has a *glaucus*-like *Pgd* allele [38], so we also developed several primers for that gene. Unfortunately, none of these primers worked. Future comparisons of *Pgd* sequences among the focal species will be useful for inferring the extent of mosaicism along the *appalachiensis* Z chromosome. We amplified all Z-linked genes with Invitrogen Platinum hot-start taq (Invitrogen Corp, Carlsbad, CA) and a touchdown protocol with annealing temperatures from 65–50°C.

We generated amplified fragment length polymorphism (AFLP) data using a Plant Mapping Kit (Applied Biosystems, Foster City, CA) and 10 primer pairs: EcoRI-ACA/MseI-CAC, EcoRI-ACA/MseI-CAG, EcoRI-ACA/MseI-CAT, EcoRI-ACA/MseI-CTC, EcoRI-ACA/MseI-CTG, EcoRI-ACT/MseI-CAC, EcoRI-ACT/MseI-CAG, EcoRI-ACT/MseI-CAT, EcoRI-ACT/MseI-CTC, and EcoRI-ACT/MseI-CTG.

### Analyses of DNA Sequence Data

We cleaned DNA sequences using Sequencher (Gene Codes Corp., Ann Arbor, MI). When available, we included reference sequences from GenBank and aligned the sequence datasets online using Multiple Sequence Comparison by Log-Expectation program (MUSCLE; http://www.ebi.ac.uk/Tools/muscle/index.html). We corrected the sequence output alignments by eye, paying special attention to indels in introns of Z-linked genes. For population genetic analyses, indels longer than one base pair were scored as a single polymorphism.

We used this dataset to analyze sequence similarity between *glaucus, canadensis* and *appalachiensis* by performing species pair-wise locus-by-locus analyses of molecular variance (AMOVAs) using Arlequin [60] to calculate fixation index (*F_ST_*) values (Figure 2B, Table S4). Given their young ages and occasional hybridization, there is considerable polymorphism but few fixed differences among the three species. Hence, we tested the genic ancestry of *appalachiensis* in relation to the parental species for polymorphisms that were strongly differentiated (p≤0.001) between *glaucus* and *canadensis* (Figure 2A). However, we used entire sequences to calculate the *F_ST_* values in Figure 2B. Specimens that were sequenced for only one gene are included in Table S3 (missing data are marked grey) and were used in calculating *F_ST_* values in Figure 2B, but excluded from Figure 2A. Genotypes here refer to nucleotide bases at specific SNP or indel polymorphisms in the genes, which can be diploid (Z-linked polymorphisms scored in males) or haploid (mtDNA, and Z-linked polymorphisms scored in females). The Z-linked genes in Figure 2A and Tables S2, S3, S4 are listed in the same order in which they occur on the *Bombyx mori* Z chromosome.

### The Use of Mitochondrial DNA to Infer the History of the W Chromosome

We wanted to determine whether *appalachiensis* inherited its W chromosome, where the gene controlling female-limited mimicry is situated, from *canadensis* or *glaucus*. Unfortunately, no W-linked molecular markers exist for these butterflies. Therefore, we used a mitochondrial gene because the W chromosome and the mitochondrial genome are both maternally inherited and thus linked in Lepidoptera. Andolfatto *et al*. [61] had attempted to use mtDNA to examine the evolutionary history of the W-linked female-limited mimetic phenotype in *glaucus.* Surprisingly, they found no association between mtDNA genealogies and mimicry, and attributed this finding to paternal leakage of mtDNA and recombination among maternal and paternal mtDNA. In fact, there are three other, potentially more likely explanations for the polyphyly of mimicry with respect to the mitochondrial gene tree: (1) multiple origins of mimicry resulting in more than one W-linked mimicry allele, (2) loss of mimicry in some individuals resulting in more than one non-mimicry allele, and (3) the presence of a low-frequency Z-linked mimicry suppressor allele in *glaucus* [26], [61]. Thus, the observed disassociation between mtDNA and mimicry does not necessarily mean disassociation between mtDNA and the W chromosome. Unfortunately, the above alternatives cannot be rigorously tested at present because the molecular identities of the mimicry and enabler genes are yet unknown. However, it is important to remember that there is overwhelming evidence from throughout the animal kingdom showing that mtDNA is almost exclusively maternally inherited [62]–[64]. Finally, it is critical to note that rare paternal inheritance of mtDNA could only influence our interpretation regarding the history of the W chromosome if it occurred during the specific time period during which *glaucus* and *canadensis* hybridized to form *appalachiensis.* If this had occurred, we would predict a mixture of *glaucus* and *canadensis* mtDNA haplotypes in *appalachiensis*, which we do not see (Figure 2A). Instead, our extensive dataset reveals that *glaucus* and *canadensis* mtDNA haplotypes are exclusively species-specific, and that of *appalachiensis* is like *glaucus* (Figure 2A). This pattern shows that paternal leakage of mtDNA has not occurred between *glaucus* and *canadensis* during the origin of *appalachiensis*. Thus, mtDNA is an informative marker for inferring the species-level ancestry of *appalachiensis*' W chromosome.

### Analyses of AFLP Data

We analyzed AFLP data using Genemapper Software Version 4.0 (Applied Biosystems, Foster City, CA). Samples were analyzed with the default bin width of 1.0 bp, and the fragment analysis range of 50–250 bps. We analyzed the AFLP data in two ways: we identified population clusters and genomic admixture among the focal species using the program STRUCTURE [65], and we constructed a phylogeny of tiger swallowtails using PAUP* [66]. We performed population clustering in STRUCTURE using three datasets: (a) focal species alone (*glaucus* (39 samples), *canadensis* (41 samples) and *appalachiensis* (38 samples); total 118 samples) (Figure S2A), (b) focal species along with laboratory-generated *glaucus/canadensis* hybrids (23 hybrid samples; total 141 samples) (Figure 3A), and (c) focal species along with laboratory-generated *glaucus/canadensis* hybrids and wild caught late flight *canadensis* individuals (30 late flight *canadensis* individuals; total 171 samples) (Figure 3C, Figure S2B).

We performed three separate STRUCTURE analyses because each was aimed at testing a different hypothesis and thus required that a different subset of samples be included. For instance, our first analysis focused only on the three focal species to determine whether *appalachiensis* exhibited evidence of admixture between *canadensis* and *glaucus* yet was genetically distinct from both of them (Figure S2A). To do this, we used a well-established approach that has been used previously to both support and refute the hypothesis of hybrid speciation [6], [67]. When admixture is present, as in the case of hybrid speciation, STRUCTURE can estimate the proportion of each individual's genome that is derived from distinct gene pools (admixture proportions). Gompert *et al*. [6] showed that a hybrid species with genomic mosaicism displayed admixture proportions split between the parental species when analyzed with the parental species at K = 2, but then formed a distinct cluster at K = 3 (just as we have shown here for *appalachiensis*) [6]. In comparison, Kronforst *et al*. [67] showed that if one does the same analysis with a species that does not appear to be a hybrid species, at K = 2 it clusters entirely with its sister species but then forms its own cluster at K = 3 [67]. Thus, we used STRUCTURE to estimate admixture proportions instead of assignment probabilities of the sampled individuals. We did this because we were asking what proportion of genome of each *appalachiensis* individual was *glaucus*- versus *canadensis*-like (which is shown by admixture proportions), not whether a particular *appalachiensis* individual was assigned to one or the other parental species with a certain probability (which is shown by assignment probabilities). Using this method, we discovered a pattern in *appalachiensis* (Figure 3) consistent with it being a hybrid species [6]. We used a similar approach for our second analysis focused on comparing the genetic signature of *appalachiensis* to lab generated *glaucus*/*canadensis* hybrids (Figure 3A), while our third analysis focused on the ancestry of the late-flight *canadensis* population (Figure 3C).

Our STRUCTURE analyses were based on 249 AFLP markers that were significantly differentiated (p≤0.05) between *glaucus* and *canadensis* as judged by *F_ST_* values from a locus-by-locus AMOVA. We analyzed each dataset in STRUCTURE with these ancestry-informative AFLP markers with 200,000 burn-in generations and 1,000,000 generations of data collection under the assumption of two, three and four admixed populations (K = 2, 3 or 4). We used STRUCTURE to estimate admixture proportions rather than assignment probabilities, and visualized the STRUCTURE outputs with the program Distruct [68] (Figure 3). Analyses based on the full AFLP dataset (polymorphisms with a minor allele frequency ≥5%) produced similar results but with less resolution (Figure S3).

For Figure 3B, we used the same 249 AFLP markers that were significantly differentiated between *glaucus* and *canadensis* (see last paragraph), did pair-wise AMOVAs among the three species, and categorized AFLP loci as: 1) “*glaucus*-like”: *appalachiensis* allele frequency is significantly different from *canadensis* but not from *glaucus*, 2) “*canadensis*-like”: *appalachiensis* allele frequency is significantly different from *glaucus* but not from *canadensis*, 3) “intermediate”: *appalachiensis* allele frequency is intermediate but not significantly different from either parental species, and 4) “different”: *appalachiensis* allele frequency is different from both parental species. The pie chart (Figure 3B) shows the number of *appalachiensis* AFLP loci that fall in the four categories, highlighting the intermediacy of *appalachiensis* with respect to *glaucus* and *canadensis*.

### Phylogenetic Methods

In order to study the larger evolutionary context of *appalachiensis*' unique hybrid phenotype, we used the AFLP dataset to resolve phylogenetic relationships between tiger swallowtails. The AFLP phylogenetic dataset included 184 individuals with *P. garamas* as the outgroup, and 1,607 polymorphic AFLP markers. Laboratory-generated *glaucus/canadensis* hybrids and suspected wild-caught *glaucus/appalachiensis* hybrids were excluded from the phylogenetic analysis. We generated a neighbor-joining tree of tiger swallowtails in PAUP* [66] (Figure 4A), with bootstrap support based on 2,000 pseudo-replicates. The distance-based neighbor-joining method was used because there is no model of evolution for AFLP data [69]–[73]. AFLPs are reliable for phylogenetic reconstruction and, in fact, provide answers consistent with other sources of data in addition to being able to resolve relationships that DNA sequence data cannot [74]–[76]. Zakharov *et al*. [40] only included *glaucus*, *canadensis*, *rutulus* and *multicaudata*, so only half of the eight tiger swallowtail species were covered in their phylogeny. Note that our relationships among those species are exactly the same as in Zakharov et al., which shows that AFLPs resolve phylogenies consistent with other sources of molecular data. The critical species for inferring the evolution of mimicry among tiger swallowtails are *alexiares* and *garcia*, which we included in our phylogeny for the first time. Several nuclear and mitochondrial genes tested were not informative in resolving relationships between some tiger swallowtail species (analysis not presented here), so fast-evolving molecular markers such as AFLPs were needed to resolve these relationships.

We mapped on this phylogeny the ecological and morphological traits (Table S6) relevant to the hybrid phenotype of *appalachiensis* using the software MacClade 4 [77]. Since phylogenetic programs do not model speciation and trait evolution on reticulated trees, for the initial mapping of traits we excluded *appalachiensis* from the tree and evolved traits from the outgroup (*P. garamas*) to *glaucus* and *canadensis*. Based on the signatures of hybrid speciation in Figure 2 and Figure 3, *appalachiensis* and its mosaic inheritance of traits from *glaucus* and *canadensis* were subsequently manually added to the tree, post-hoc.

### Estimating Divergence Times and Gene Flow

We used our DNA sequence data and the program IMa2 [78]–[81] to estimate divergence times (Figure 5) and historical gene flow (Figure 6) among *appalachiensis*, *glaucus* and *canadensis*. This coalescent-based method uses comparative DNA sequence data and an “Isolation with Migration” model of population divergence to estimate model parameters such as effective population sizes, divergence times, and bi-directional migration rates. While IMa2 can analyze more than two populations at a time, it requires populations to be related by a specified bifurcating tree. Since we were testing the predicted hybrid origin of *appalachiensis*, which does not follow a bifurcating mode of speciation, we analyzed our data as three pair-wise comparisons among the three focal species. For each analysis, we ran IMa2 with 100 Metropolis-coupled chains with a 150,000-step burn-in followed by 20 million steps of data collection. An important requirement of the IMa2 method is that there is free recombination among sequenced loci: loci do not necessarily have to be on separate chromosomes but they should not be too close to one another. Since six of our seven sequenced loci were on the Z chromosome, and they spanned the length of that chromosome, we satisfied this requirement by using data from only three genes for these analyses: *Kettin* and *PAH*, which are on opposite ends of the Z chromosome, and the mitochondrial gene *COI*. Another requirement of IMa2 is that there is no recombination within loci. We satisfied this requirement by trimming the sequence dataset to a region with no evidence of recombination using the program IMgc Online [82]. We used this trimmed dataset with three genes for estimating both divergence times and gene flow. Converting parameter estimates to units such as time in years since divergence or population migration rates (2Nm) requires mutation rates for each gene. We estimated these by comparing sequences to the within tiger swallowtail outgroup, *P. multicaudata*. Average mtDNA divergence between *multicaudata* and the three focal species was 3.17%. Using previous *COI* divergence estimate of 1.15% per lineage per million years [83], we estimated that *P. multicaudata* split from the common ancestor of our focal species approximately 1.4 million years ago. This date differs from that estimated by Zakharov *et al*. [40] for all of *Papilio*, but our date may be more accurate since it was estimated specifically for tiger swallowtails. However, the actual dates are less important for our purpose since the relevant findings from the IMa2 analysis were that: (a) *appalachiensis* originated much more recently compared to the time when *glaucus* and *canadensis* split from one another (Figure 5), and (b) *appalachiensis* split from both *glaucus* and *canadensis* around the same time (Figure 5).

A similar analysis was done to estimate divergence times between *glaucus*, *canadensis* and late-flight *canadensis* (Figure S4).

### Comparisons of Linkage Disequilibrium

We estimated the extent of linkage disequilibrium across both our AFLP and Z-linked DNA sequence data and compared this among populations. For each dataset (AFLPs or concatenated Z-linked gene sequences) we calculated pair-wise associations among all polymorphisms using the exact test for linkage disequilibrium implemented in Arlequin. We summarized these results by calculating the average percentage of polymorphisms that were significantly (*p*≤0.01) associated with each other (Table 1) and then compared them among species using an ANOVA.

### Comparisons of Species-Specific Polymorphisms

We used our DNA sequence dataset to calculate the number of species-specific polymorphisms for each of our focal species (Table 2). We calculated species-specific polymorphisms as the proportion of genetic polymorphisms unique to one species, out of the total number of polymorphisms (including shared and species-specific) seen among all the sequenced samples of that species. We used the software DnaSP [84] to count the total number of polymorphisms and the subset that were species-specific for each species at each gene. We then compared the average proportion of species-specific polymorphisms among species with an ANOVA. We also performed a similar analysis comparing the proportion of species-specific haplotypes rather than polymorphisms, and found similar results (Table 2).

## Supporting Information

Figure S1 Distribution of wingspan (representing body size) among *glaucus*, *appalachiensis* and *canadensis* (mean±SD): *glaucus*: 57±6.11 mm, n = 45; *appalachiensis*: 57±3.11 mm, n = 45; *canadensis*: 47±2.04 mm, n = 23. ANOVA: *F_(2,110)_* =  45.588; *p*<0.0001.
(TIF)

Figure S2 STRUCTURE analysis comparing (A) *appalachiensis* with the parental species, and (B) *appalachiensis* with lab hybrids and late flight *canadensis*.
(TIF)

Figure S3 STRUCTURE analysis with all polymorphic AFLP markers with a minor allele frequency > 5%.
(TIF)

Figure S4 Estimated divergence times between *glaucus*, *canadensis* and late flight *canadensis*. Dates of divergence estimated by IMa2 are: (a) late flight *canadensis* and *glaucus*: approximately 400,000 years ago, (b) late flight *canadensis* and *canadensis*: approximately zero years ago.
(TIF)

Figure S5 Localities and states where specimens used in this study were collected. For each locality, the number of specimens collected of each focal species is shown. can  =  *Papilio canadensis*, late flight can  =  *canadensis* late flight, app  =  *P. appalachiensis*, gla  =  *P. glaucus*, and gla-app suspected hybrids  =  individuals phenotypically intermediate between *glaucus* and *appalachiensis*, hence suspected to be hybrids between the two species.
(TIF)

Table S1 Specimens used in this work.
(PDF)

Table S2 Genes, primer sequences, and lengths of aligned sequences used in this work.
(DOC)

Table S3 Complete genotype table for Figure 2.
(PDF)

Table S4 A comparison of locus-by-locus AMOVA among genes and species. *F_ST_* values and their statistical significance are shown for each gene and each species pair-wise comparison. Also shown are the numbers of loci (i.e., genetic polymorphisms) that were significantly different either at p≤0.05 or at p≤0.001. This table has been condensed into Figure 2B.
(DOC)

Table S5 Pair-wise population differentiation among tiger swallowtails based on AFLP data. Overall *F_ST_*: 0.16, p<0.001. Population pair-wise *F_ST_*.
(DOC)

Table S6 Ecological and morphological differentiation between tiger swallowtails.
(DOC)
