# Supplementary figures and images for: Sex Chromosome Mosaicism and Hybrid Speciation among Tiger Swallowtail Butterflies
Source: PLoS Genet. 2011 Sep 8;7(9):e1002274. doi: 10.1371/journal.pgen.1002274 (PMC3169544; doi:10.1371/journal.pgen.1002274)

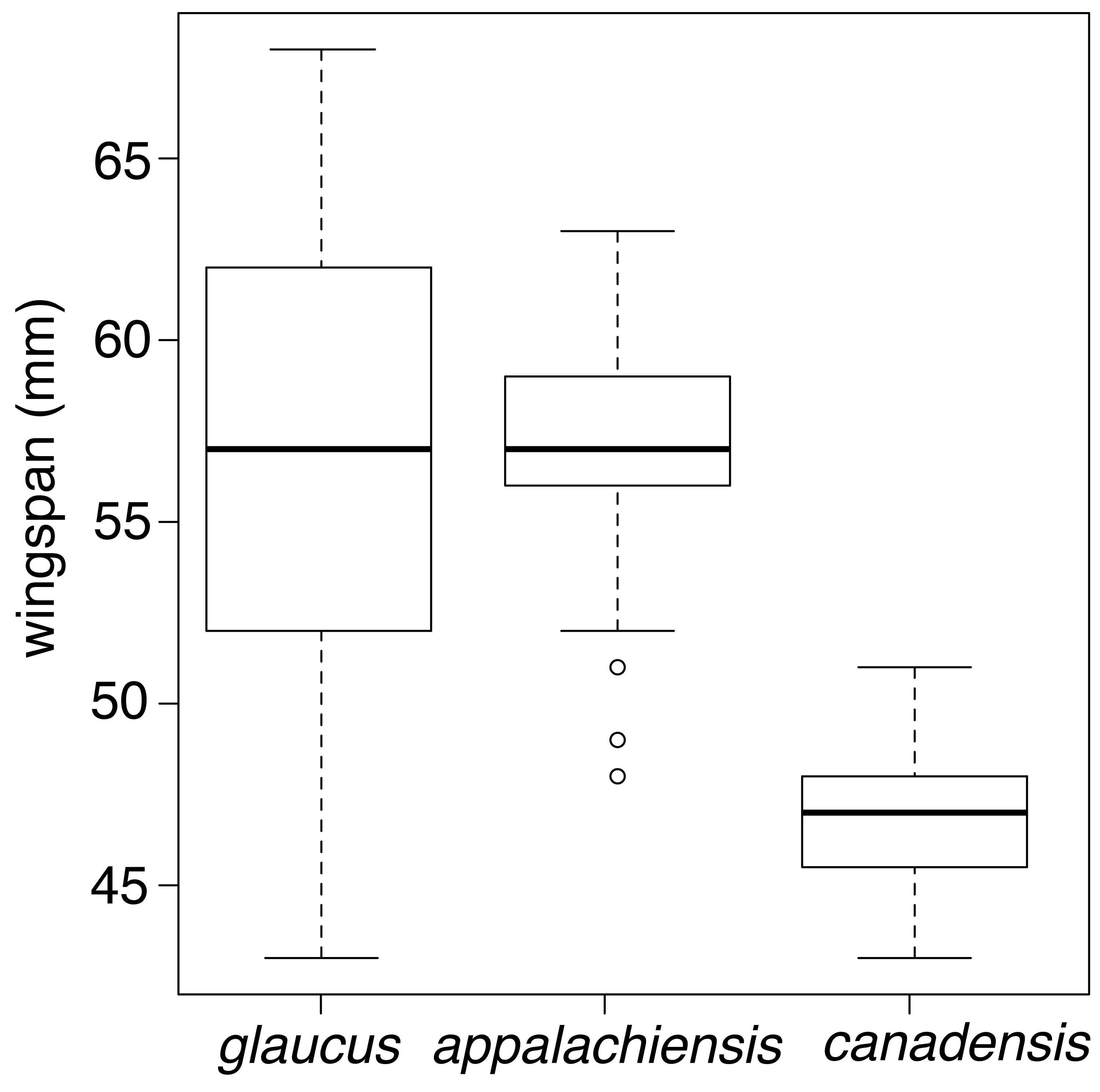

Supplement: Figure S1 — Distribution of wingspan (representing body size) among glaucus, appalachiensis and canadensis (mean±SD): glaucus: 57±6.11 mm, n = 45; appalachiensis: 57±3.11 mm, n = 45; canadensis: 47±2.04 mm, n = 23. ANOVA: F(2,110) = 45.588; p<0.0001. (TIF) [file pgen.1002274.s001.tif]

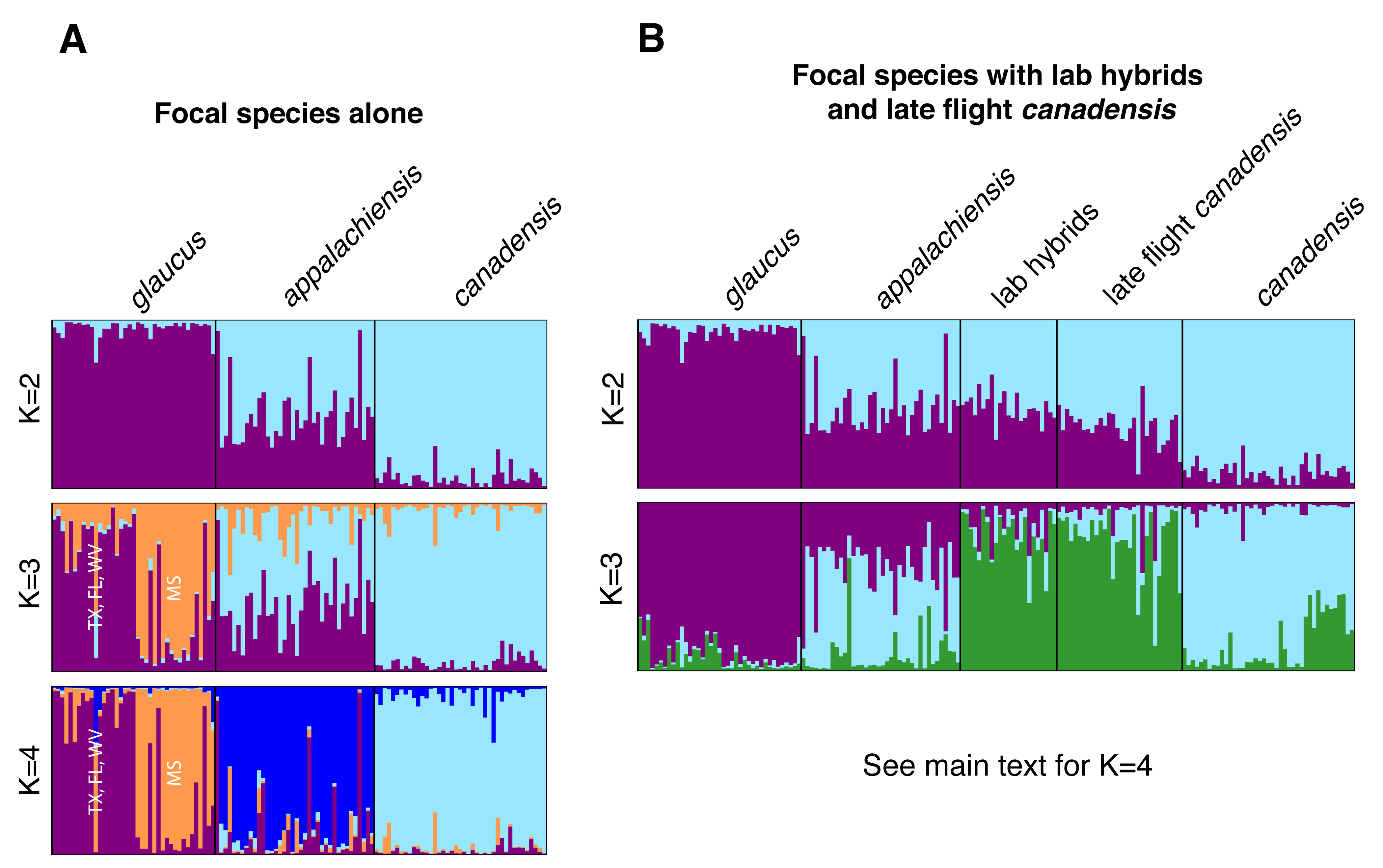

Supplement: Figure S2 — STRUCTURE analysis comparing (A) appalachiensis with the parental species, and (B) appalachiensis with lab hybrids and late flight canadensis. (TIF) [file pgen.1002274.s002.tif]

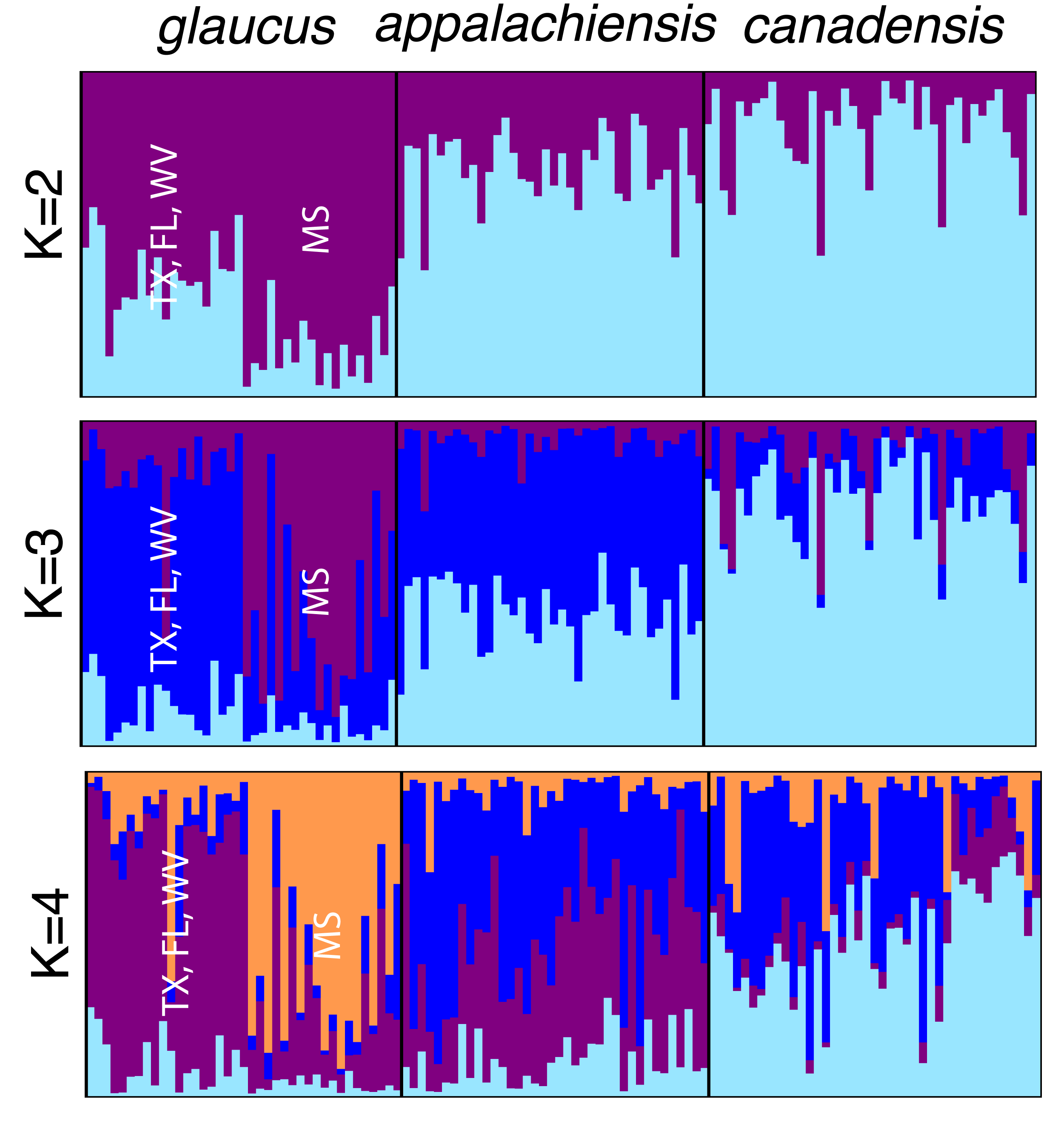

Supplement: Figure S3 — STRUCTURE analysis with all polymorphic AFLP markers with a minor allele frequency > 5%. (TIF) [file pgen.1002274.s003.tif]

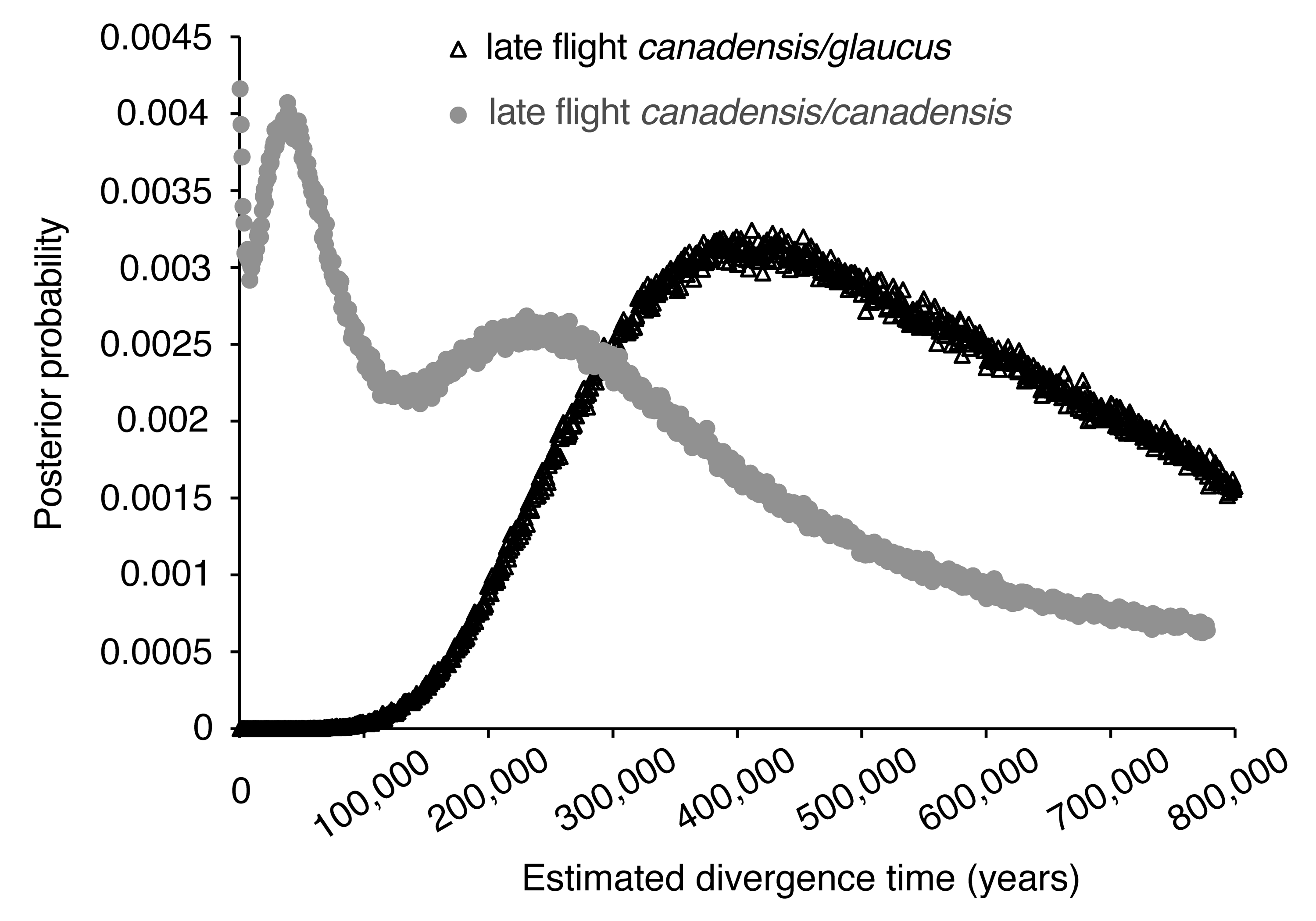

Supplement: Figure S4 — Estimated divergence times between glaucus, canadensis and late flight canadensis. Dates of divergence estimated by IMa2 are: (a) late flight canadensis and glaucus: approximately 400,000 years ago, (b) late flight canadensis and canadensis: approximately zero years ago. (TIF) [file pgen.1002274.s004.tif]

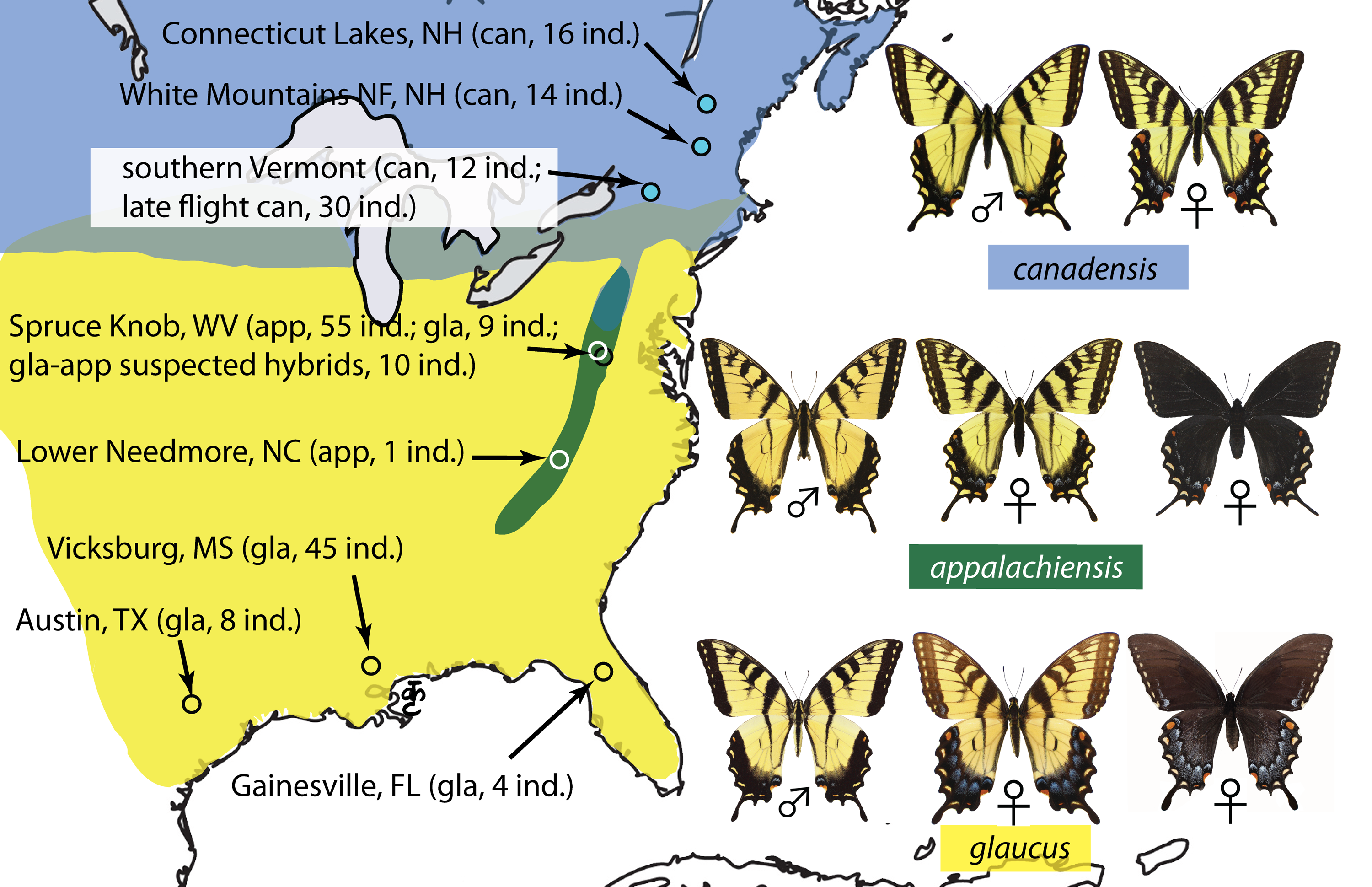

Supplement: Figure S5 — Localities and states where specimens used in this study were collected. For each locality, the number of specimens collected of each focal species is shown. can = Papilio canadensis, late flight can = canadensis late flight, app = P. appalachiensis, gla = P. glaucus, and gla-app suspected hybrids = individuals phenotypically intermediate between glaucus and appalachiensis, hence suspected to be hybrids between the two species. (TIF) [file pgen.1002274.s005.tif]
