# Supplementary material for: Sex Chromosome Mosaicism and Hybrid Speciation among Tiger Swallowtail Butterflies
Source: PLoS Genet. 2011 Sep 8;7(9):e1002274. doi: 10.1371/journal.pgen.1002274 (PMC3169544; doi:10.1371/journal.pgen.1002274)
Supplement: Table S1 — Specimens used in this work. (PDF) [file pgen.1002274.s006.pdf]

Supporting Information:

Table S1: Specimens of the *Papilio glaucus* species group and the outgroup (*P. garamas*) used in this work. All specimens, except where mentioned otherwise, were collected by Krushnamegh Kunte. The "*appalachiensis/glaucus*" specimens were phenotypically intermediate between *glaucus* and *appalachiensis*; hence, suspected to be hybrids.

Specimen	Species	Sex	Collection information
AA070	*garamas*	Female	Parque Chipinque, Monterrey, Mexico. 2006/06.
AA838	*garamas*	Female	Balcon de Montezuma, Tamaulipas, Mexico. 2006/09.
AA873	*garamas*	Male	Canon de la Libertad, Tamaulipas, Mexico. 2006/09.
AA875	*garamas*	Male	Canon de la Libertad, Tamaulipas, Mexico. 2006/09.
AA876	*garamas*	Male	Canon de la Libertad, Tamaulipas, Mexico. 2006/09.
AA878	*garamas*	Female	Canon de la Libertad, Tamaulipas, Mexico. 2006/09.
AA880	*garamas*	Female	Canon de la Libertad, Tamaulipas, Mexico. 2006/09.
AA050	*multicaudata*	Male	Austin, Texas, USA. 2006/03.
AA051	*multicaudata*	Male	Austin, Texas, USA. 2006/03.
AA054	*multicaudata*	Male	Austin, Texas, USA. 2006/03.
AA055	*multicaudata*	Male	Austin, Texas, USA. 2006/03.
AA059	*multicaudata*	Female	Austin, Texas, USA. 2006/03.
AA079	*multicaudata*	Male	Austin, Texas, USA. 2006/03.
AA083	*multicaudata*	Female	Austin, Texas, USA. 2006/04.
AA089	*multicaudata*	Female	Austin, Texas, USA. 2006/04.
AA369	*multicaudata*	Female	Austin, Texas, USA. 2006/06.
AA422	*rutulus*	Female	Roosevelt National Forest, Colorado, USA. 2006/07.
AA425	*rutulus*	Female	Roosevelt National Forest, Colorado, USA. 2006/07.
AA435	*rutulus*	Male	Roosevelt National Forest, Colorado, USA. 2006/07.
AA437	*rutulus*	Male	Roosevelt National Forest, Colorado, USA. 2006/07.
AA438	*rutulus*	Male	Roosevelt National Forest, Colorado, USA. 2006/07.
AA440	*rutulus*	Male	Roosevelt National Forest, Colorado, USA. 2006/07.
AA441	*rutulus*	Male	Roosevelt National Forest, Colorado, USA. 2006/07.
AA444	*rutulus*	Female	Roosevelt National Forest, Colorado, USA. 2006/07.
AA420	*eurymedon*	Female	Roosevelt National Forest, Colorado, USA. 2006/07.
AA447	*eurymedon*	Male	Greenhorn Mountains, California, USA. 2006/06. Coll: Ken Davenport.
AA448	*eurymedon*	Male	Greenhorn Mountains, California, USA. 2006/06. Coll: Ken Davenport.
AA449	*eurymedon*	Male	Greenhorn Mountains, California, USA. 2006/06. Coll: Ken Davenport.
AA450	*eurymedon*	Female	Santa Barbara Canyons, California, USA. 2006/05. Coll: Ken Davenport.
AA451	*eurymedon*	Female	Santa Barbara Canyons, California, USA. 2006/05. Coll: Ken Davenport.
AA453	*eurymedon*	Male	Santa Barbara Canyons, California, USA. 2006/05. Coll: Ken Davenport.
AA454	*eurymedon*	Male	Prefund Canyon, California, USA. 1994/04. Coll: Ken Davenport.
AA562	*alexiares*	Male	Sierra del Doctor, Queretaro, Mexico. 1999/04. Coll: Rafael and Alonso Turrent.
AA563	*alexiares*	Male	Sierra del Doctor, Queretaro, Mexico. 2002/04. Coll: Rafael and Alonso Turrent.
AA025	*garcia*	Female	Parque Chipinque, Monterrey, Mexico. 2005/09.
AA071	*garcia*	Female, black	Parque Chipinque, Monterrey, Mexico. 2006/06.
AA072	*garcia*	Male	Parque Chipinque, Monterrey, Mexico. 2006/06.
AA073	*garcia*	Male	Parque Chipinque, Monterrey, Mexico. 2006/06.
AA077	*garcia*	Female, black	Parque Chipinque, Monterrey, Mexico. 2006/06.
AA870	*garcia*	Male	Canon de la Libertad, Tamaulipas, Mexico. 2006/09. Coll: Irmi Garcia.
AA026	*glaucus*	Male	Gainesville, Florida, USA. 2005/06.
AA027	*glaucus*	Male	Gainesville, Florida, USA. 2005/06.
AA028	*glaucus*	Female, black	Gainesville, Florida, USA. 2005/06.
AA049	*glaucus*	Male	Austin, Texas, USA. 2006/03.
AA052	*glaucus*	Male	Austin, Texas, USA. 2006/03.
AA053	*glaucus*	Male	Austin, Texas, USA. 2006/03.
AA056	*glaucus*	Female, black	Austin, Texas, USA. 2006/03.
AA062	*glaucus*	Female, black	Austin, Texas, USA. 2006/04.
AA066	*glaucus*	Female, yellow	Austin, Texas, USA. 2006/05.
AA084	*glaucus*	Female, black	Austin, Texas, USA. 2006/04.
AA280	*glaucus*	Male	Spruce Knob, West Virginia, USA. 2006/05.
AA304	*glaucus*	Female, black	Spruce Knob, West Virginia, USA. 2006/05.
AA314	*glaucus*	Female, yellow	Spruce Knob, West Virginia, USA. 2006/05.
AA316	*glaucus*	Female, yellow	Spruce Knob, West Virginia, USA. 2006/05.
AA319	*glaucus*	Female, black	Spruce Knob, West Virginia, USA. 2006/05.
AA321	*glaucus*	Female, black	Spruce Knob, West Virginia, USA. 2006/05.
AA325	*glaucus*	Female, black	Spruce Knob, West Virginia, USA. 2006/05.
AA339	*glaucus*	Female, black	Spruce Knob, West Virginia, USA. 2006/05.
AA365	*glaucus*	Female, black	Spruce Knob, West Virginia, USA. 2006/05.
AA366	*glaucus*	Male	Austin, Texas, USA. 2006/06.
AA367	*glaucus*	Female, yellow	Gainesville, Florida, USA. 2006/06.
AA512	*glaucus*	Male	Vicksburg, Mississippi, USA. 2006/08. Coll: Ricky Patterson.
AA518	*glaucus*	Male	Vicksburg, Mississippi, USA. 2006/08. Coll: Ricky Patterson.
AA531	*glaucus*	Male	Vicksburg, Mississippi, USA. 2006/08. Coll: Ricky Patterson.
AA533	*glaucus*	Male	Vicksburg, Mississippi, USA. 2006/08. Coll: Ricky Patterson.
AA537	*glaucus*	Female, black	Vicksburg, Mississippi, USA. 2006/08. Coll: Ricky Patterson.
AA538	*glaucus*	Female, black	Vicksburg, Mississippi, USA. 2006/08. Coll: Ricky Patterson.

Specimen	Species	Sex	Collection information
AA540	*glaucus*	Female, yellow	Vicksburg, Mississippi, USA. 2006/08. Coll: Ricky Patterson.
AA543	*glaucus*	Female, black	Vicksburg, Mississippi, USA. 2006/08. Coll: Ricky Patterson.
AA544	*glaucus*	Female, black	Vicksburg, Mississippi, USA. 2006/08. Coll: Ricky Patterson.
AA545	*glaucus*	Female, black	Vicksburg, Mississippi, USA. 2006/08. Coll: Ricky Patterson.
AA546	*glaucus*	Female, black	Vicksburg, Mississippi, USA. 2006/08. Coll: Ricky Patterson.
AA547	*glaucus*	Female, black	Vicksburg, Mississippi, USA. 2006/08. Coll: Ricky Patterson.
AA548	*glaucus*	Female, yellow	Vicksburg, Mississippi, USA. 2006/08. Coll: Ricky Patterson.
AA549	*glaucus*	Female, black	Vicksburg, Mississippi, USA. 2006/08. Coll: Ricky Patterson.
AA551	*glaucus*	Female, black	Vicksburg, Mississippi, USA. 2006/08. Coll: Ricky Patterson.
AA552	*glaucus*	Female, yellow	Vicksburg, Mississippi, USA. 2006/08. Coll: Ricky Patterson.
AA553	*glaucus*	Female, yellow	Vicksburg, Mississippi, USA. 2006/08. Coll: Ricky Patterson.
AA554	*glaucus*	Female, yellow	Vicksburg, Mississippi, USA. 2006/08. Coll: Ricky Patterson.
AA555	*glaucus*	Female, black	Vicksburg, Mississippi, USA. 2006/08. Coll: Ricky Patterson.
AA556	*glaucus*	Female, black	Vicksburg, Mississippi, USA. 2006/08. Coll: Ricky Patterson.
AA557	*glaucus*	Female, black	Vicksburg, Mississippi, USA. 2006/08. Coll: Ricky Patterson.
AA558	*glaucus*	Female, black	Vicksburg, Mississippi, USA. 2006/08. Coll: Ricky Patterson.
AA559	*glaucus*	Female, black	Vicksburg, Mississippi, USA. 2006/08. Coll: Ricky Patterson.
AA560	*glaucus*	Female, yellow	Vicksburg, Mississippi, USA. 2006/08. Coll: Ricky Patterson.
AB002	*glaucus*	Female, black	Vicksburg, Mississippi, USA. 2006/08. Coll: Ricky Patterson.
AB003	*glaucus*	Female, black	Vicksburg, Mississippi, USA. 2006/08. Coll: Ricky Patterson.
AB004	*glaucus*	Female, yellow	Vicksburg, Mississippi, USA. 2006/08. Coll: Ricky Patterson.
AB005	*glaucus*	Female, black	Vicksburg, Mississippi, USA. 2006/08. Coll: Ricky Patterson.
AB006	*glaucus*	Female, black	Vicksburg, Mississippi, USA. 2006/08. Coll: Ricky Patterson.
AB007	*glaucus*	Female, black	Vicksburg, Mississippi, USA. 2006/08. Coll: Ricky Patterson.
AB008	*glaucus*	Female, black	Vicksburg, Mississippi, USA. 2006/08. Coll: Ricky Patterson.
AB009	*glaucus*	Female, black	Vicksburg, Mississippi, USA. 2006/08. Coll: Ricky Patterson.
AB010	*glaucus*	Female, black	Vicksburg, Mississippi, USA. 2006/08. Coll: Ricky Patterson.
AB011	*glaucus*	Female, yellow	Vicksburg, Mississippi, USA. 2006/08. Coll: Ricky Patterson.
AB012	*glaucus*	Female, yellow	Vicksburg, Mississippi, USA. 2006/08. Coll: Ricky Patterson.
AB013	*glaucus*	Female, yellow	Vicksburg, Mississippi, USA. 2006/08. Coll: Ricky Patterson.
AB014	*glaucus*	Female, yellow	Vicksburg, Mississippi, USA. 2006/08. Coll: Ricky Patterson.
AB015	*glaucus*	Female, yellow	Vicksburg, Mississippi, USA. 2006/08. Coll: Ricky Patterson.
AB016	*glaucus*	Female, yellow	Vicksburg, Mississippi, USA. 2006/08. Coll: Ricky Patterson.
AB017	*glaucus*	Female, yellow	Vicksburg, Mississippi, USA. 2006/08. Coll: Ricky Patterson.
AB018	*glaucus*	Female, yellow	Vicksburg, Mississippi, USA. 2006/08. Coll: Ricky Patterson.
AB019	*glaucus*	Female, yellow	Vicksburg, Mississippi, USA. 2006/08. Coll: Ricky Patterson.
AB020	*glaucus*	Female, yellow	Vicksburg, Mississippi, USA. 2006/08. Coll: Ricky Patterson.
AB021	*glaucus*	Female, yellow	Vicksburg, Mississippi, USA. 2006/08. Coll: Ricky Patterson.
AB022	*glaucus*	Female, yellow	Vicksburg, Mississippi, USA. 2006/08. Coll: Ricky Patterson.
AA165	*appalachiensis*	Female, black	Lower Needmore, North Carolina, USA. 2005/06.
AA263	*appalachiensis*	Male	Spruce Knob, West Virginia, USA. 2006/05.
AA267	*appalachiensis*	Male	Spruce Knob, West Virginia, USA. 2006/05.
AA281	*appalachiensis*	Male	Spruce Knob, West Virginia, USA. 2006/05.
AA282	*appalachiensis*	Male	Spruce Knob, West Virginia, USA. 2006/05.
AA285	*appalachiensis*	Male	Spruce Knob, West Virginia, USA. 2006/05.
AA291	*appalachiensis*	Male	Spruce Knob, West Virginia, USA. 2006/05.
AA298	*appalachiensis*	Female, black	Spruce Knob, West Virginia, USA. 2006/05.
AA299	*appalachiensis*	Female, black	Spruce Knob, West Virginia, USA. 2006/05.
AA301	*appalachiensis*	Female, yellow	Spruce Knob, West Virginia, USA. 2006/05.
AA302	*appalachiensis*	Female, yellow	Spruce Knob, West Virginia, USA. 2006/05.
AA303	*appalachiensis*	Female, yellow	Spruce Knob, West Virginia, USA. 2006/05.
AA306	*appalachiensis*	Female, yellow	Spruce Knob, West Virginia, USA. 2006/05.
AA307	*appalachiensis*	Female, yellow	Spruce Knob, West Virginia, USA. 2006/05.
AA309	*appalachiensis*	Female, yellow	Spruce Knob, West Virginia, USA. 2006/05.
AA310	*appalachiensis*	Female, yellow	Spruce Knob, West Virginia, USA. 2006/05.
AA311	*appalachiensis*	Female, yellow	Spruce Knob, West Virginia, USA. 2006/05.
AA312	*appalachiensis*	Female, yellow	Spruce Knob, West Virginia, USA. 2006/05.
AA313	*appalachiensis*	Female, yellow	Spruce Knob, West Virginia, USA. 2006/05.
AA315	*appalachiensis*	Female, yellow	Spruce Knob, West Virginia, USA. 2006/05.
AA318	*appalachiensis*	Female, black	Spruce Knob, West Virginia, USA. 2006/05.
AA322	*appalachiensis*	Female, aberrant	Spruce Knob, West Virginia, USA. 2006/05.
AA323	*appalachiensis*	Female, black	Spruce Knob, West Virginia, USA. 2006/05.
AA326	*appalachiensis*	Female, black	Spruce Knob, West Virginia, USA. 2006/05.
AA329	*appalachiensis*	Female, yellow	Spruce Knob, West Virginia, USA. 2006/05.
AA333	*appalachiensis*	Female, black	Spruce Knob, West Virginia, USA. 2006/05.
AA335	*appalachiensis*	Female, black	Spruce Knob, West Virginia, USA. 2006/05.
AA346	*appalachiensis*	Female, yellow	Spruce Knob, West Virginia, USA. 2006/05.
AA348	*appalachiensis*	Female, black	Spruce Knob, West Virginia, USA. 2006/05.
AA349	*appalachiensis*	Female, aberrant	Spruce Knob, West Virginia, USA. 2006/05.
AA350	*appalachiensis*	Male	Spruce Knob, West Virginia, USA. 2006/05.
AA351	*appalachiensis*	Female, yellow	Spruce Knob, West Virginia, USA. 2006/05.
AA353	*appalachiensis*	Female, yellow	Spruce Knob, West Virginia, USA. 2006/05.

Specimen	Species	Sex	Collection information
AA354	*appalachiensis*	Female, yellow	Spruce Knob, West Virginia, USA. 2006/05.
AA355	*appalachiensis*	Female, yellow	Spruce Knob, West Virginia, USA. 2006/05.
AA356	*appalachiensis*	Female, yellow	Spruce Knob, West Virginia, USA. 2006/05.
AA358	*appalachiensis*	Female, aberrant	Spruce Knob, West Virginia, USA. 2006/05.
AA360	*appalachiensis*	Female, black	Spruce Knob, West Virginia, USA. 2006/05.
AA361	*appalachiensis*	Female, aberrant	Spruce Knob, West Virginia, USA. 2006/05.
AA364	*appalachiensis*	Female, black	Spruce Knob, West Virginia, USA. 2006/05.
AA370	*appalachiensis*	Female, yellow	Spruce Knob, West Virginia, USA. 2006/06.
AA456	*appalachiensis*	Male	Spruce Knob, West Virginia, USA. 2006/06.
AA457	*appalachiensis*	Female	Spruce Knob, West Virginia, USA. 2008/06.
AA458	*appalachiensis*	Female	Spruce Knob, West Virginia, USA. 2008/06.
AA459	*appalachiensis*	Female	Spruce Knob, West Virginia, USA. 2008/06.
AA460	*appalachiensis*	Female	Spruce Knob, West Virginia, USA. 2008/06.
AA461	*appalachiensis*	Female, aberrant	Spruce Knob, West Virginia, USA. 2008/06.
AA462	*appalachiensis*	Female	Spruce Knob, West Virginia, USA. 2008/06.
AA464	*appalachiensis*	Male	Spruce Knob, West Virginia, USA. 2008/06.
AA465	*appalachiensis*	Male	Spruce Knob, West Virginia, USA. 2008/06.
AA466	*appalachiensis*	Male	Spruce Knob, West Virginia, USA. 2008/06.
AA467	*appalachiensis*	Female	Spruce Knob, West Virginia, USA. 2008/06.
AA468	*appalachiensis*	Male	Spruce Knob, West Virginia, USA. 2008/06.
AA469	*appalachiensis*	Male	Spruce Knob, West Virginia, USA. 2008/06.
AA470	*appalachiensis*	Male	Spruce Knob, West Virginia, USA. 2008/06.
AA336	*appalachiensis*	Female, black	Spruce Knob, West Virginia, USA. 2006/05.
AA300	*appalachiensis/glaucus*	Female, yellow	Spruce Knob, West Virginia, USA. 2006/05.
AA308	*appalachiensis/glaucus*	Female, yellow	Spruce Knob, West Virginia, USA. 2006/05.
AA317	*appalachiensis/glaucus*	Female, black	Spruce Knob, West Virginia, USA. 2006/05.
AA320	*appalachiensis/glaucus*	Female, black	Spruce Knob, West Virginia, USA. 2006/05.
AA324	*appalachiensis/glaucus*	Female, black	Spruce Knob, West Virginia, USA. 2006/05.
AA327	*appalachiensis/glaucus*	Female, black	Spruce Knob, West Virginia, USA. 2006/05.
AA328	*appalachiensis/glaucus*	Female, black	Spruce Knob, West Virginia, USA. 2006/05.
AA337	*appalachiensis/glaucus*	Female, black	Spruce Knob, West Virginia, USA. 2006/05.
AA341	*appalachiensis/glaucus*	Female, yellow	Spruce Knob, West Virginia, USA. 2006/05.
AA359	*appalachiensis/glaucus*	Female, aberrant	Spruce Knob, West Virginia, USA. 2006/05.
MA05	*canadensis* (May)	Female	Vermont, USA. Lab-reared by Matthew Aardema.
MA53	*canadensis* (May)	Male	Vermont, USA. Lab-reared by Matthew Aardema.
MA54	*canadensis* (May)	Female	Vermont, USA. Lab-reared by Matthew Aardema.
MA56	*canadensis* (May)	Female	Vermont, USA. Lab-reared by Matthew Aardema.
MA57	*canadensis* (May)	Female	Vermont, USA. Lab-reared by Matthew Aardema.
MA58	*canadensis* (May)	Female	Vermont, USA. Lab-reared by Matthew Aardema.
MA59	*canadensis* (May)	Male	Vermont, USA. Lab-reared by Matthew Aardema.
MA60	*canadensis* (May)	Female	Vermont, USA. Lab-reared by Matthew Aardema.
MA61	*canadensis* (May)	Male	Vermont, USA. Lab-reared by Matthew Aardema.
MA64	*canadensis* (May)	Female	Vermont, USA. Lab-reared by Matthew Aardema.
MA65	*canadensis* (May)	Female	Vermont, USA. Lab-reared by Matthew Aardema.
MA66	*canadensis* (May)	Male	Vermont, USA. Lab-reared by Matthew Aardema.
AA377	*canadensis* (June)	Male	White Mountains NF, New Hampshire, USA. 2006/06.
AA378	*canadensis* (June)	Female	White Mountains NF, New Hampshire, USA. 2006/06.
AA379	*canadensis* (June)	Female	White Mountains NF, New Hampshire, USA. 2006/06.
AA380	*canadensis* (June)	Female	White Mountains NF, New Hampshire, USA. 2006/06.
AA386	*canadensis* (June)	Male	White Mountains NF, New Hampshire, USA. 2006/06.
AA387	*canadensis* (June)	Male	White Mountains NF, New Hampshire, USA. 2006/06.
AA389	*canadensis* (June)	Male	White Mountains NF, New Hampshire, USA. 2006/06.
AA390	*canadensis* (June)	Male	White Mountains NF, New Hampshire, USA. 2006/06.
AA391	*canadensis* (June)	Female	White Mountains NF, New Hampshire, USA. 2006/06.
AA392	*canadensis* (June)	Female	White Mountains NF, New Hampshire, USA. 2006/06.
AA393	*canadensis* (June)	Female	White Mountains NF, New Hampshire, USA. 2006/06.
AA394	*canadensis* (June)	Male	White Mountains NF, New Hampshire, USA. 2006/06.
AA400	*canadensis* (June)	Male	White Mountains NF, New Hampshire, USA. 2006/06.
AA401	*canadensis* (June)	Male	White Mountains NF, New Hampshire, USA. 2006/06.
AA402	*canadensis* (June)	Male	Connecticut Lakes, New Hampshire, USA. 2006/06.
AA403	*canadensis* (June)	Male	Connecticut Lakes, New Hampshire, USA. 2006/06.
AA404	*canadensis* (June)	Male	Connecticut Lakes, New Hampshire, USA. 2006/06.
AA405	*canadensis* (June)	Male	Connecticut Lakes, New Hampshire, USA. 2006/06.
AA407	*canadensis* (June)	Male	Connecticut Lakes, New Hampshire, USA. 2006/06.
AA408	*canadensis* (June)	Male	Connecticut Lakes, New Hampshire, USA. 2006/06.
AA409	*canadensis* (June)	Male	Connecticut Lakes, New Hampshire, USA. 2006/06.
AA410	*canadensis* (June)	Male	Connecticut Lakes, New Hampshire, USA. 2006/06.
AA411	*canadensis* (June)	Male	Connecticut Lakes, New Hampshire, USA. 2006/06.
AA412	*canadensis* (June)	Male	Connecticut Lakes, New Hampshire, USA. 2006/06.
AA413	*canadensis* (June)	Male	Connecticut Lakes, New Hampshire, USA. 2006/06.
AA414	*canadensis* (June)	Male	Connecticut Lakes, New Hampshire, USA. 2006/06.
AA415	*canadensis* (June)	Male	Connecticut Lakes, New Hampshire, USA. 2006/06.

**Specimen**	**Species**	**Sex**	**Collection information**
AA416	*canadensis* (June)	Male	Connecticut Lakes, New Hampshire, USA. 2006/06.
AA418	*canadensis* (June)	Female	Connecticut Lakes, New Hampshire, USA. 2006/06.
AA419	*canadensis* (June)	Female	Connecticut Lakes, New Hampshire, USA. 2006/06.
MA19	*canadensis* (July)	Male	Vermont, USA. 2008/07. Coll: Matthew Aardema.
MA20	*canadensis* (July)	Male	Vermont, USA. 2008/07. Coll: Matthew Aardema.
MA21	*canadensis* (July)	Male	Vermont, USA. 2008/07. Coll: Matthew Aardema.
MA22	*canadensis* (July)	Male	Vermont, USA. 2008/07. Coll: Matthew Aardema.
MA23	*canadensis* (July)	Male	Vermont, USA. 2008/07. Coll: Matthew Aardema.
MA24	*canadensis* (July)	Male	Vermont, USA. 2008/07. Coll: Matthew Aardema.
MA25	*canadensis* (July)	Male	Vermont, USA. 2008/07. Coll: Matthew Aardema.
MA26	*canadensis* (July)	Male	Vermont, USA. 2008/07. Coll: Matthew Aardema.
MA27	*canadensis* (July)	Male	Vermont, USA. 2008/07. Coll: Matthew Aardema.
MA28	*canadensis* (July)	Male	Vermont, USA. 2008/07. Coll: Matthew Aardema.
MA29	*canadensis* (July)	Male	Vermont, USA. 2008/07. Coll: Matthew Aardema.
MA30	*canadensis* (July)	Male	Vermont, USA. 2008/07. Coll: Matthew Aardema.
MA31	*canadensis* (July)	Male	Vermont, USA. 2008/07. Coll: Matthew Aardema.
MA32	*canadensis* (July)	Male	Vermont, USA. 2008/07. Coll: Matthew Aardema.
MA33	*canadensis* (July)	Male	Vermont, USA. 2008/07. Coll: Matthew Aardema.
MA34	*canadensis* (July)	Male	Vermont, USA. 2008/07. Coll: Matthew Aardema.
MA35	*canadensis* (July)	Male	Vermont, USA. 2008/07. Coll: Matthew Aardema.
MA36	*canadensis* (July)	Male	Vermont, USA. 2008/07. Coll: Matthew Aardema.
MA37	*canadensis* (July)	Male	Vermont, USA. 2008/07. Coll: Matthew Aardema.
MA38	*canadensis* (July)	Male	Vermont, USA. 2008/07. Coll: Matthew Aardema.
MA40	*canadensis* (July)	Male	Vermont, USA. 2008/07. Coll: Matthew Aardema.
MA41	*canadensis* (July)	Male	Vermont, USA. 2008/07. Coll: Matthew Aardema.
MA42	*canadensis* (July)	Male	Vermont, USA. 2008/07. Coll: Matthew Aardema.
MA43	*canadensis* (July)	Male	Vermont, USA. 2008/07. Coll: Matthew Aardema.
MA44	*canadensis* (July)	Male	Vermont, USA. 2008/07. Coll: Matthew Aardema.
MA45	*canadensis* (July)	Male	Vermont, USA. 2008/07. Coll: Matthew Aardema.
MA46	*canadensis* (July)	Male	Vermont, USA. 2008/07. Coll: Matthew Aardema.
MA47	*canadensis* (July)	Male	Vermont, USA. 2008/07. Coll: Matthew Aardema.
MA48	*canadensis* (July)	Male	Vermont, USA. 2008/07. Coll: Matthew Aardema.
MA55	*canadensis* (July)	Male	Vermont, USA. Lab-reared by Matthew Aardema.
MA01	*glaucus/canadensis F1*	Male	Vermont *canadensis* x Ohio *glaucus*, by Matthew Aardema and Mark Scriber.
MA02	*glaucus/canadensis F1*	Male	Vermont *canadensis* x Ohio *glaucus*, by Matthew Aardema and Mark Scriber.
MA03	*glaucus/canadensis F2*	Male	Hybrid F1 crossed among themselves, by Matthew Aardema and Mark Scriber.
MA04	*glaucus/canadensis F2*	Female	Hybrid F1 crossed among themselves, by Matthew Aardema and Mark Scriber.
MA06	*glaucus/canadensis F1*	Male	Vermont *canadensis* x Ohio *glaucus*, by Matthew Aardema and Mark Scriber.
MA07	*glaucus/canadensis F1*	Female	Vermont *canadensis* x Ohio *glaucus*, by Matthew Aardema and Mark Scriber.
MA08	*glaucus/canadensis F1*	Male	Vermont *canadensis* x Ohio *glaucus*, by Matthew Aardema and Mark Scriber.
MA09	*glaucus/canadensis F1*	Male	Vermont *canadensis* x Ohio *glaucus*, by Matthew Aardema and Mark Scriber.
MA10	*glaucus/canadensis F1*	Male	Vermont *canadensis* x Ohio *glaucus*, by Matthew Aardema and Mark Scriber.
MA11	*glaucus/canadensis F1*	Male	Vermont *canadensis* x Ohio *glaucus*, by Matthew Aardema and Mark Scriber.
MA12	*glaucus/canadensis F1*	Male	Vermont *canadensis* x Ohio *glaucus*, by Matthew Aardema and Mark Scriber.
MA13	*glaucus/canadensis F1*	Male	Alaska *canadensis* x Ohio *glaucus*, by Matthew Aardema and Mark Scriber.
MA14	*glaucus/canadensis F2*	Female	Hybrid F1 crossed among themselves, by Matthew Aardema and Mark Scriber.
MA15	*glaucus/canadensis F1*	Male	Alaska *canadensis* x Ohio *glaucus*, by Matthew Aardema and Mark Scriber.
MA16	*glaucus/canadensis F1*	Male	Alaska *canadensis* x Ohio *glaucus*, by Matthew Aardema and Mark Scriber.
MA17	*glaucus/canadensis F1*	Male	Alaska *canadensis* x Ohio *glaucus*, by Matthew Aardema and Mark Scriber.
MA18	*glaucus/canadensis F1*	Male	Alaska *canadensis* x Ohio *glaucus*, by Matthew Aardema and Mark Scriber.
MA49	*glaucus/canadensis F1*	Male	Vermont *canadensis* x Ohio *glaucus*, by Matthew Aardema and Mark Scriber.
MA50	*glaucus/canadensis F1*	Male	Vermont *canadensis* x Ohio *glaucus*, by Matthew Aardema and Mark Scriber.
MA51	*glaucus/canadensis F1*	Male	Alaska *canadensis* x Ohio *glaucus*, by Matthew Aardema and Mark Scriber.
MA52	*glaucus/canadensis F1*	Male	Vermont *canadensis* x Ohio *glaucus*, by Matthew Aardema and Mark Scriber.
MA62	*glaucus/canadensis F1*	Male	Vermont *canadensis* x Ohio *glaucus*, by Matthew Aardema and Mark Scriber.
MA63	*glaucus/canadensis F1*	Male	Alaska *canadensis* x Ohio *glaucus*, by Matthew Aardema and Mark Scriber.
