# Supplementary material for: Sex Chromosome Mosaicism and Hybrid Speciation among Tiger Swallowtail Butterflies
Source: PLoS Genet. 2011 Sep 8;7(9):e1002274. doi: 10.1371/journal.pgen.1002274 (PMC3169544; doi:10.1371/journal.pgen.1002274)
Supplement: Table S2 — Genes, primer sequences, and lengths of aligned sequences used in this work. (DOC) [file pgen.1002274.s007.doc]

**Table S2:** Genes, primer sequences and lengths of aligned sequences used in this work.

| **Gene** | **Primer** | **Length** |
| --- | --- | --- |
| *Cytochrome oxydase I* (*COI*; mitochondrial)^1^ | Jerry (forward): CAACATTTATTTTGATTTTTTGG  Pat (reverse): TCCATTACATATAATCTGCCATATTAG  PatII (reverse): TCCAATGCACTAATCTGCCATATTA | 839bp |
| *Kettin* (*Ket*; Z-linked)^2^ | Kettin_f1: GAATTTGAGAAACCTATATT  Kettin_f2: CATGTGCATTTAGAAGCTCA  Kettin_r1: GGTTAACGAAACTCCATTCT  Kettin_r2: GTTTATCACCAGTACACCTT | 1,128bp |
| *Tyrosine hydroxylase*  (*TH*; Z-linked)^3^ | TH_540f: TGAAGAGGAAGTTATTCTGC  TH_1260r: TGNGTNGAYTGRAANACNCGRAANGC | 762bp |
| *Triosephosphate isomerase* (*Tpi*; Z-linked)^2^ | TPI_f1: TTGGTGAAAAAGATGACCTG  TPI_r1: GCAATYACTTTCATGCCTGA | 995bp |
| *Period* (*Per*; Z-linked)^2^ | Per_f1: CGACTCCATTCTTCTCAGCG  Per_r1: ATGAATAGAACTGGTTTWGT | 894bp |
| *Lactate dehydrogenase* (*Ldh*; Z-linked)^2^ | LDH_f1: ATGATGCGTCGCTTTACGTT  LDH_r1: GATGATCTCGTAGGCGCTCT | 460bp |
| *Phenylalanine hydro-xylase* (*PAH*; Z-linked)^3^ | Papilio_PAH_875f: CACCATTCAAAGCCACTATACAC  Papilio_PAH_1062r: AATCCAAATTCTACTGTGAACCA | 1,171bp |

^1^: primer sequences from Nazari V., Zakharov E.V., Sperling F.A.H. (2007) Phylogeny, historical biogeography, and taxonomic ranking of Parnassiinae (Lepidoptera, Papilionidae) based on morphology and seven genes. *Mol. Phylogen. Evol.* 42: 131-156.

^2^: newly designed specific primers, based on Putnam A.S., Scriber J.M., Andolfatto P. (2007) Discordant divergence times among Z-chromosome regions between two ecologically distinct swallowtail butterfly species. *Evolution* 61: 912-927.

^3^: newly designed primers.
