# Supplementary material for: Sex Chromosome Mosaicism and Hybrid Speciation among Tiger Swallowtail Butterflies
Source: PLoS Genet. 2011 Sep 8;7(9):e1002274. doi: 10.1371/journal.pgen.1002274 (PMC3169544; doi:10.1371/journal.pgen.1002274)
Supplement: Table S4 — A comparison of locus-by-locus AMOVA among genes and species. FST values and their statistical significance are shown for each gene and each species pair-wise comparison. Also shown are the numbers of loci (i.e., genetic polymorphisms) that were significantly different either at p≤0.05 or at p≤0.001. This table has been condensed into Figure 2B. (DOC) [file pgen.1002274.s009.doc]

**Table S4:** A comparison of locus-by-locus AMOVA among genes and species. *F_ST_* values and their statistical significance are shown for each gene and each species pair-wise comparison. Also shown are the numbers of loci (i.e., genetic polymorphisms) that were significantly different either at p≤0.05 or at p≤0.001. This table has been condensed into Fig. 2B.

|  | **All three species** | ***glaucus* vs. *canadensis*** | ***glaucus* vs. *appalachiensis*** | ***canadensis* vs. *appalachiensis*** |
| --- | --- | --- | --- | --- |
| ***COI*** | *F_ST_* = 0.772 (p<0.001) | *F_ST_* = 0.824 (p≤0.001)  15 loci: p≤0.05  11 loci: p≤0.001 | *F_ST_* = 0.048 (p=0.026)  1 locus: p=0.047 | *F_ST_* = 0.844  (p≤0.001)  13 loci: p≤0.05  10 loci: p≤0.001 |
| ***Ket*** | *F_ST_* = 0.333 (p<0.001) | *F_ST_* = 0.416 (p≤0.001)  18 loci: p≤0.05  8 loci: p≤0.001 | *F_ST_* = 0.504 (p≤0.001)  25 loci: p≤0.05  8 loci: p≤0.001 | *F_ST_* = 0.019 (p=0.182)  10 loci: p≤0.05  0 loci: p≤0.001 |
| ***TH*** | *F_ST_* = 0.532 (p<0.001) | *F_ST_* = 0.694 (p≤0.001)  15 loci: p≤0.05  9 loci: p≤0.001 | *F_ST_* = 0.536 (p≤0.001)  15 loci: p≤0.05  9 loci: p≤0.001 | *F_ST_* = 0.064 (p=0.01)  5 loci: p≤0.05  0 loci: p≤0.001 |
| ***Tpi*** | *F_ST_* = 0.220 (p<0.001) | *F_ST_* = 0.273 (p≤0.001)  46 loci: p≤0.05  27 loci: p≤0.001 | *F_ST_* = 0.341 (p≤0.001)  39 loci: p≤0.05  14 loci: p≤0.001 | *F_ST_* = 0.018 (p=0.235)  11 loci: p≤0.05  0 loci: p≤0.001 |
| ***Per*** | *F_ST_* = 0.161 (p<0.001) | *F_ST_* = 0.267 (p≤0.001)  12 loci: p≤0.05  5 loci: p≤0.001 | *F_ST_* = 0.081 (p≤0.006)  7 loci: p≤0.05  5 loci: p≤0.001 | *F_ST_* = 0.087 (p=0.016)  2 loci: p≤0.05  0 loci: p≤0.001 |
| ***Ldh*** | *F_ST_* = 0.179 (p<0.001) | *F_ST_* = 0.25 (p≤0.001)  30 loci: p≤0.05  17 loci: p≤0.001 | *F_ST_* = 0.217 (p≤0.001)  69 loci: p≤0.05  4 loci: p≤0.001 | *F_ST_* = 0.038 (p=0.108)  11 loci: p≤0.05  0 loci: p≤0.001 |
| ***PAH*** | *F_ST_* = 0.320 (p<0.001) | *F_ST_* = 0.485 (p≤0.001)  64 loci: p≤0.05  41 loci: p≤0.001 | *F_ST_* = 0.201 (p≤0.001)  38 loci: p≤0.05  16 loci: p≤0.001 | *F_ST_* = 0.105 (p=0.001)  26 loci: p≤0.05  1 locus: p≤0.001 |
