# Supplementary material for: Sex Chromosome Mosaicism and Hybrid Speciation among Tiger Swallowtail Butterflies
Source: PLoS Genet. 2011 Sep 8;7(9):e1002274. doi: 10.1371/journal.pgen.1002274 (PMC3169544; doi:10.1371/journal.pgen.1002274)
Supplement: Table S5 — Pair-wise population differentiation among tiger swallowtails based on AFLP data. Overall FST: 0.16, p<0.001. Population pair-wise FST. (DOC) [file pgen.1002274.s010.doc]

**Table S5:** Pair-wise population differentiation among tiger swallowtails based on AFLP data. Overall *F_ST_*: 0.16, p<0.001. Population pair-wise *F_ST_*:

1 2 3 4 5 6 7 8 9 10 11 12 13 14

*garamas* (7)^a^ 1 0.000

*multicaudata* (8) 2 0.586^b^  0.000

*rutulus* (8) 3 0.556 0.440 0.000

*eurymedon* (7) 4 0.539 0.418 0.215 0.000

*alexiares* (2) 5 0.632* 0.472* 0.397* 0.381* 0.000

*garcia* (7) 6 0.623 0.518 0.444 0.441 0.236* 0.000

*glaucus* (TX: 8, FL: 4, WV: 8) 7 0.418 0.307 0.249 0.237 0.172* 0.241 0.000

*glaucus* (MS: 19) 8 0.394 0.300 0.261 0.245 0.132* 0.246 0.053 0.000

*appalachiensis* (38) 9 0.409 0.306 0.253 0.246 0.180* 0.247 0.037 0.075 0.000

*canadensis*: Vermont: May (12) 10 0.434 0.340 0.245 0.258 0.182* 0.271 0.065 0.087 0.051 0.000

*canadensis*: NH: late June (29) 11 0.396 0.305 0.232 0.237 0.178* 0.256 0.080 0.087 0.041 0.028 0.000

*canadensis*: late flight: July (30) 12 0.400 0.313 0.233 0.241 0.175* 0.238 0.059 0.098 0.056 0.009* 0.056 0.000

*glaucus-can.* hybrids (23) 13 0.411 0.319 0.235 0.248 0.184* 0.246 0.042 0.100 0.057 0.032 0.070 0.028 0.000

*glaucus-appy.* hybrids (8) 14 0.472 0.351 0.291 0.277 0.205* 0.298 0.026 0.053 0.041 0.086 0.089 0.060 0.058 0.000

^a^: Numbers in parentheses are samples sizes. ^b^: All pairwise *F_ST_* values are significant at p<0.001, except those marked with * are p<0.05.
