# Supplementary material for: Sex Chromosome Mosaicism and Hybrid Speciation among Tiger Swallowtail Butterflies
Source: PLoS Genet. 2011 Sep 8;7(9):e1002274. doi: 10.1371/journal.pgen.1002274 (PMC3169544; doi:10.1371/journal.pgen.1002274)
Supplement: Table S6 — Ecological and morphological differentiation between tiger swallowtails. (DOC) [file pgen.1002274.s011.doc]

**Table S6:** Ecological and morphological differentiation between tiger swallowtails.

|  | **Sexual**  **dimorphism** | **Female**  **dimorphism** | **Batesian mimicry** | **Female black form**  **Enabler** | **Female yellow form** |
| --- | --- | --- | --- | --- | --- |
| ***garamas* (outgroup)** | dimorphic | monomorphic | mimetic | ? | absent |
| ***multicaudata*** | monomorphic | monomorphic | non-mimetic | suppressor | present |
| ***eurymedon*** | monomorphic | monomorphic | non-mimetic | suppressor | present |
| ***rutulus*** | monomorphic | monomorphic | non-mimetic | suppressor | present |
| ***alexiares*** | monomorphic | monomorphic | non-mimetic | ? | present |
| ***garcia*** | dimorphic | monomorphic | mimetic | enabler | absent |
| ***glaucus*** | dimorphic | dimorphic | mimetic | enabler | present |
| ***appalachiensis*** | dimorphic | dimorphic | mimetic | enabler | present |
| ***canadensis*** | monomorphic | monomorphic | non-mimetic | suppressor | present |
