# Supplementary material for: Sex Chromosome Mosaicism and Hybrid Speciation among Tiger Swallowtail Butterflies
Source: PLoS Genet. 2011 Sep 8;7(9):e1002274. doi: 10.1371/journal.pgen.1002274 (PMC3169544; doi:10.1371/journal.pgen.1002274)
Supplement: Table S3 — Complete genotype table for Figure 2. (PDF) [file pgen.1002274.s008.pdf]

Table S3. Complete genotype table for all the specimens used in this work. Missing data are shown by grey. Late flight *canadensis* individuals are marked by orange. Indels are represented by base/-.

Group	Gene	Base	AA026FLGIM	AA027FLGIM	AA028FLGIFB	AA056TXGIFB	AA062TXGIFB	AA066TXGIFY	AA084TXGIFB	AA316WVGIFY	AA319WVGIFB	AA321WVGIFB	AA325WVGIFB	AA339WVGIFB	AA365WVGIFB	AA366TXGIM	AA367FLGIFY	AA512GIM	AA537GlaFB	AA538GlaFB	AA540GlaFY	AA543GlaFB	AA544GlaFB	AA545GlaFB	AA546GlaFB	AA547GlaFB	AA548GlaFY	AA549GlaFB	AA551GlaFB	AA552GlaFY	AA553GlaFY	AA554GlaFY
			glaucus	*glaucus*	*glaucus*	*glaucus*	*glaucus*	*glaucus*	*glaucus*	*glaucus*	*glaucus*	*glaucus*	*glaucus*	*glaucus*	*glaucus*	*glaucus*	*glaucus*	*glaucus*	*glaucus*	*glaucus*	*glaucus*	*glaucus*	*glaucus*	*glaucus*	*glaucus*	*glaucus*	*glaucus*	*glaucus*	*glaucus*	*glaucus*	*glaucus*	*glaucus*
mt gene	COI	18	C	C	C	C	C	C	C	C	C		C	C	C	C	C	C	C	C	C	C	C	C	C	C	C	C	C	C	C	C
	COI	93	G	G	G	G	G	G	G	G	G		G	G	G	G	G	G	G	G	G	G	G	G	G	G	G	G	G	G	G	G
	COI	393	T	T	T	T	T	T	T	T	T		T	T	T	T	T	T	T	T	T	T	T	T	T	T	T	T	T	T	T	T
	COI	402	T	T	T	T	T	T	T	T	T		T	T	T	T	T	T	T	T	T	T	T	T	T	T	T	T	T	T	T	T
	COI	489	C	C	C	C	C	C	C	C	C		C	C	C	C	C	C	C	C	C	C	C	C	C	C	C	C	C	C	C	C
	COI	558	A	G	G	A	G	G	G	G	A		A	G	G	G	G	G	G	G	G	G	G	A	A	G	G	G	G	G	G	G
	COI	627	T	T	T	T	T	T	T	T	T		T	T	T	T	T	T	T	T	T	T	T	T	T	T	T	T	T	T	T	T
	COI	642	C	C	C	C	C	C	C	C	C		C	C	C	C	C	C	C	C	C	C	C	C	C	C	C	C	C	C	C	C
	COI	687	G	G	G	G	G	G	G	G	G		G	G	G	G	G	G	G	G	G	G	G	G	G	G	G	G	G	G	G	G
	COI	768	A	A	A	A	A	A	A	A	A		A	A	A	A	A	A	A	A	A	A	A	A	A	A	A	A	A	A	A	A
	COI	789	C	C	C	C	C	C	C	C	C		C	C	C	C	C	C	C	T	C	C	C	C	C	C	C	C	T	C	C	C
Z-linked genes	Kettin	72	A	A	A	A	A	A	A	A	A	A	A		A		A	A			A									A	A	A
	Kettin	78	T	T	T	T	T	T	T	T	T	T	T		T		T	T			T									T	T	T
	Kettin	148	T	T	T	T	T	T	T	T	T	T	T		T		T	T			T									T	T	T
	Kettin	157	T	T	T	T	T	T	T	T	T	T	T		T		T	T			T									T	T	T
	Kettin	215	G	G	G	G	G	G	G	G	G	G	G		G		G	G			G									G	G	G
	Kettin	401	T	T	T	T	T	T	T	T	T	T	T		T		T	T			T									T	T	T
	Kettin	683	C	C	C	C	C	C	C	C		C	C		C		C	C			C									C	C	C
	Kettin	1040	G	G	G	G	G	G	G	G		G	G		G		G	G			G									G	G	G
	TH	65	A	A	A	A	A	A	A	A	A	A	A	A	A	A		A			A									A	A	A
	TH	161	G	G	G	G	G	G	G	G	G	G	G	G	G	G		G			G									G	G	G
	TH	176	T	T	G	T	T	G	T	G	T	T	T	G	G	T		K			G									G	G	T
	TH	183	A	A	A	A	A	A	A	A	A	A	A	A	A	A		A			A									A	A	A
	TH	228	G	G	G	G	G	G	G	G	G	G	G	A	G	G		G			A									G	A	G
	TH	341	T	T	C	T	C	T	T	T	T	T	T	T	T	Y		T			T									C	T	C
	TH	458	C	C	C	C	C	C	C	C	C	C	T	T	C	C		Y			T									C	T	T
	TH	476	C	C	C	C	T	T	C	C	C	C	C	C	C	C		C			C									C	C	C
	TH	530	G	G	G	G	G	G	G	G	G	G	G	G	G	G		G			G									G	G	G
	Tpi	80	–	T		A	A	A	T	A	A	A	T	A	A	A	A	T			A									T	A	
	Tpi	83	–	T		T	T	T	T	T	T	T	T	T	T	T	T	T			T									T	T	
	Tpi	106	–	T		–	–	–	T	–	–	–	T	–	–	–	–	T			–									T	–	
	Tpi	131	–	T		–	–	–	T	–	–	–	T	–	–	–	–	T			–									T	–	
	Tpi	132	–	T		–	–	–	T	–	–	–	T	–	–	–	–	T			–									T	–	
	Tpi	134	–	C		–	–	–	C	–	–	–	C	–	–	–	–	C			–									C	–	
	Tpi	145	–	T		–	–	–	T	–	–	–	T	–	–	–	–	T			–									T	–	
	Tpi	151	–	T		–	–	–	T	–	–	–	T	–	–	–	–	T			–									T	–	
	Tpi	153	–	T		–	–	–	T	–	–	–	T	–	–	–	–	T			–									T	–	
	Tpi	156	–	G		–	–	–	G	–	–	–	G	–	–	–	–	G			–									G	–	
	Tpi	163	–	–		–	–	–	–	–	–	–	–	–	–	–	–	–			–									–	–	
	Tpi	171	–	A		–	–	–	T	–	–	–	T	–	–	–	–	T			–									T	–	
	Tpi	172	–	A		–	–	–	A	–	–	–	A	–	–	–	–	A			–									A	–	
	Tpi	173	–	A		–	–	–	A	–	–	–	A	–	–	–	–	A			–									A	–	
	Tpi	194	–	T		–	–	–	T	–	–	–	T	–	–	–	–	T			–									T	–	
	Tpi	216	–	T		–	–	–	T	–	–	–	T	–	–	–	–	T			–									T	–	
	Tpi	217	–	A		–	–	–	A	–	–	–	A	–	–	–	–	A			–									A	–	
	Tpi	219	–	–		–	–	–	–	–	–	–	–	–	–	–	–	–			–									–	–	
	Tpi	220	–	–		–	–	–	–	–	–	–	–	–	–	–	–	–			–									–	–	
	Tpi	222	?/-	T		–	–	–	T	–	–	–	T	–	–	–	–	T			–									T	–	
	Tpi	231	–	G		–	–	–	G	–	–	–	G	–	–	–	–	G			?									G		
	Tpi	265	C	C		C	C	C	C	C	C	C	C	C	C	C	C	C			C									C	C	
	Tpi	287	G	G		G	G	G	G	G	G	G	G	G	G	G	G	G			G									G	G	
	Tpi	318	G	G		G	G	G	A	G	A	G	A	A	A	G	G	A			A									A	G	
	Tpi	322	T	T		T	T	T	T	T	T	T	T	T	T	T	T	T			T									T	T	
	Tpi	335	C	C		C	C	C	C	C	C	C	C	C	C	C	C	C			C									C	C	
	Tpi	339	A	T		A	A	A	T	A	T	A	T	T	T	A	A	T			T									T	A	
	Period	32	?	?	G	?	G	G				G	C		G		G	G			G									G	G	G
	Period	165	G	G	G	G	G	G	G	G	G	G	–	G	G	G	G	G			G									G	G	G
	Period	172	T	T	T	T	–	T	T	T	T	T	–	T	T	T	T	T			T									T	T	T
	Period	435	T	T	T	T	T	?	T	T	T	T	C	T	T	T	T	T			T									T	T	T
	Period	465	G	?	?	G	G	G	G	G	G	G		G	G	G	G	G			G									G	G	G
	Ldh	30	C	T	T	C	C		T	T		T	T	?	C		C	T			C									C	C	
	Ldh	96	T	C	C	C	C		C	C		C	C	C	C		C	C			C									T	T	
	Ldh	126	W	T	A	T	A		A	T		T	A	A	T		T	A			T									T	T	
	Ldh	141	A	A	A	A	A		A	T		A	A	A	A		A	A			A									A	A	
	Ldh	142	G	G	G	G	G		G	C		C	G	G	G		G	G			G									G	G	
	Ldh	144	A	A	A	A	A		A	G		G	A	A	A		A	A			A									A	A	
	Ldh	146	C	C	C	C	C		C	T		C	C	C	C		C	C			C									C	C	
	Ldh	207	G	C	G	G	G		C	C		C	G	C	G		C	C			G									C	G	
	Ldh	219	Y	C	C	C	C		T	C		C	C	C	T		C	T			C									C	T	
	Ldh	223	T	T	T	T	T		T	G		T	T	T	T		T	T			T									T	T	
	Ldh	237	C	T	T	T	C		C	C		C	T	C	C		C	C			T									C	C	
	Ldh	243	A	A	–	–	A		A	A		A	–	A	A		A	A			–									A	A	
	Ldh	244	M	C	–	–	C		C	C		C	–	A	C		C	C			–									A	C	
	Ldh	262	G	G	–	–	G		G	G		G	–	G	G		G	G			–									G	G	
	Ldh	263	T	T	–	–	T		T	T		T	–	T	T		T	T			–									T	T	
	Ldh	398	C	M	C	C	C		C	M		C	C	C	C		C	C			C									C	C	
	Ldh	416	A	C	C	A	A		C	C		C	C	A	C		C	C			A									C	C	
	PAH	35	C	C	T	T	C	T	C	C	C	C	C	C	C	Y	C	T			T									T	T	C
	PAH	102	C	Y	C	C	C	T	T	C	T	C	C	C	C	Y	C	C			T									T	C	C
	PAH	131	C	C	T	T	T	C	C	T	C	T	C	C	T	Y	C	Y			T									C	C	T
	PAH	257	G	R	A	A	G	A	A	A	G	A	G	A	A	R	A	G			A									A	A	A
	PAH	286	G	G	?	G	G	G	G	G	A	G	G	G	G	G	G	G			G									G	G	G
	PAH	304	C	C	C	C	A	A	A	C	T	A	C	C	C	A	C	M			C									C	C	A
	PAH	310	C	C	C	C	C	C	C	C	A	C	C	C	C	C	C	C			C									C	C	C
	PAH	311	C	C	C	C	C	C	C	C	T	C	C	C	C	C	C	C			C									C	C	C
	PAH	321	C	–	–	–	–	C	C	–	C	–	C	–	–	C	C	C/[illegible]			–									C	–	C
	PAH	327	?	A	A	A	A	G	G	A	G	A	?	A	A	G/[illegible]	?	R			A										A	G
	PAH	368	T	C	C	C	T	T	C	T	C	C	T	C	C	Y	T	Y			T									T	C	T
	PAH	470	R	S	A	C	A	A	C	A	A	A	A	A	C	C	A	C			A									A	C	A
	PAH	521	–		A	A	–	A	–	A	A	–	–	A	A	A	A	A/-			–									–	A	–
	PAH	524	W		C	A	T	A	T	A	A	T	T	A	A	W	A	W			T									T	A	T
	PAH	526	T		C	C	T	C	T	C	C	T	T	C	C	C	C	Y			T									T	C	T
	PAH	529	Y		C	C	C	C	C	C	T	C	C	T	C	Y	C	C			C									C	C	C
	PAH	536	W		A	A	T	A	T	A	A	T	T	A	A	A	A	W			T									T	A	T
	PAH	541	A/–		–	–	A	A	A	–	–	A	A	–	–	–	–	A/-			A									G	–	A
	PAH	546	G		T	C	G	G	G	T	T	G	G	T	C	C	C	S			G									G	C	G
	PAH	553	S		G	C	G	G	G	C	G	G	G	C	C	C	C	S			G									G	C	G
	PAH	559	K		T	T	G	G	G	T	T	G	G	T	T	T	T	K			G									G	T	G
	PAH	561	C		T	C	C	C	C	C	T	C	C	C	C	C	C	C			C									C	C	C
	PAH	575	M		A	C	A	A	A	C	A	A	A	C	C	C	C	M			A									A	C	A
	PAH	578	S		A	C	G	G	G	C	A	G	G	C	C	C	C	S			G									G	C	G
	PAH	579	G		A	G	G	G	G	G	A	G	G	G	G	G	G	G			G									G	G	G
	PAH	586	C/–		C	C	T	T	T	C	C	T	T	C	C	C	C	Y			T									T	C	T
	PAH	588	A/?		A	A	G	G	G	A	A	G	G	A	A	A	A	R			G									G	A	G
	PAH	590	C/?		T	T	C	C	C	T	T	C	C	T	T	T	T	Y			C									C	T	C
	PAH	600	C/?		C	T	C	C	G	C	T	G	G	C	T	C	C	Y			G									C	C	C
	PAH	638	T		T	C	C	C	C	T	T	C	C	C	C	C	C	C			C									C	C	
	PAH	658	T	T	T	T	T	T	T	T	C	T	T	T	T	T	T	T			T									T	T	T
	PAH	677	G	G	G	G	G	G	G	G	A	G	G	G	G	G	G	G			G									G	G	G
	PAH	682	A	A	A	G	A	A	A	A	A	G	G	A	G	G	G	R			G									A	G	A
	PAH	702	A	A	A	A	A		A	A	G	A	A	A	A	A	A	A			A									A	A	A
	PAH	716	C	C	C	T	C	C	C	C	T	C	C	C	T	C	C	C			C									C	C	C
	PAH	720	A	A	A	A	A	?	A	A	T	A	A	A	A	A	A	A			A									A	A	A
	PAH	738	C	M	C	C	G	C	A	C	T	A	C	A	C	C	C	M			C									C	G	A
	PAH	742	–	–	–	–	–	–	–	–	A	–	–	–	–	–	–	–			–									–	–	–
	PAH	748	C	C	C	C	C	C	C	C	T	C	C	C	C	C	C	C			C									C	C	C
	PAH	753	T	T	T	T	T	T	T	T	A	T	T	T	T	T	T	T			T									T	T	T
	PAH	772	C	?	C	C	C	C	C	C		C	C	C	C	C					C									C	C	C

Group	Gene	Base	AA555GlaFB	AA556GlaFB	AA557GlaFB	AA558GlaFB	AA559GlaFB	AA560GlaFY	AB002GlaFB	AB003GlaFB	AB004GlaFY	AB005GlaFY	AB006GlaFB	AB007GlaFB	AB008GlaFB	AB009GlaFB	AB010GlaFB	AB011GlaFY	AB012GlaFY	AB013GlaFY	AB014GlaFY	AB015GlaFY	AB016GlaFY	AB017GlaFY	AB018GlaFY	AB019GlaFY	AB020GlaFY	AB021GlaFY	AB022GlaFY
			glaucus	*glaucus*	*glaucus*	*glaucus*	*glaucus*	*glaucus*	*glaucus*	*glaucus*	*glaucus*	*glaucus*	*glaucus*	*glaucus*	*glaucus*	*glaucus*	*glaucus*	*glaucus*	*glaucus*	*glaucus*	*glaucus*	*glaucus*	*glaucus*	*glaucus*	*glaucus*	*glaucus*	*glaucus*	*glaucus*	*glaucus*
mt gene	COI	18	C	C	C	C	C	C	C	C	C		C	C	C	C	C	C	C	C	C		C	C	C	C	C	C	C
	COI	93	G	G	G	A	G	G	G	G	G		G	G	G	G	G	G	G	G	G		G	G	G	G	G	G	G
	COI	393	T	T	T	T	T	T	T	T	T		T	T	T	T	T	T	T	T	T		T	T	T	T	T	T	T
	COI	402	T	T	T	T	T	T	T	T	T		T	T	T	T	T	T	T	T	T		T	T	T	T	T	T	T
	COI	489	C	C	C	C	C	C	C	C	C		C	C	C	C	C	C	C	C	C		C	C	C	C	C	C	C
	COI	558	A	A	G	G	G	G	G	G	G		G	G	G	G	G	G	G	A	G		G	G	G	G	A	A	G
	COI	627	T	T	T	T	T	T	T	T	T		T	T	T	T	T	T	T	T	T		T	T	T	T	T	T	T
	COI	642	C	C	C	C	C	C	C	C	C		C	C	C	C	C	C	C	C	C		C	C	C	C	C	C	C
	COI	687	G	G	G	G	G	G	G	G	G		G	G	G	G	G	G	G	G	G		G	G	G	G	G	G	G
	COI	768	A	A	A	A	A	A	A	A	A		A	A	A	A	A	A	A	A	A		A	A	A	A	A	A	A
	COI	789	C	C	C	C	C	C	C	C	C		C	C	C	C	C	C	C	C	C		C	C	C	C	C	C	C
Z-linked genes	TH	65	A	A	A	A	A	A	A	A	A	A						A	A	A	A	A							
	TH	161	G	G	G	G	G	G	G	G	G	G						G	G	G	G	G							
	TH	176	G	T	T	G	G	T	G	T	G	T						G	G	G	G	G							
	TH	183	A	A	A	A	A	A	A	A	A	A						A	A	A	A	A							
	TH	228	G	G	G	G	G	G	G	G	G	G						G	G	G	G	G							
	TH	341	C	T	T	T	C	T	T	T	C	T						C	T	T	C	T							
	TH	458	C	T	C	C	T	T	C	T	T	C						C	C	C	C	T							
	TH	476	C	C	C	C	C	C	C	C	C	C						C	C	C	C	C							
	TH	530	G	G	G	G	G	G	G	G	G	G						G	G	G	G	G							

Group	Gene	Base	AA165ApFB	AA291ApM	AA298ApFB	AA299ApFB	AA300ApFY	AA301ApFY	AA302ApFY	AA303ApFY	AA306ApFY	AA307ApFY	AA308ApFY	AA309ApFY	AA311ApFY	AA312ApFY	AA313ApFY	AA315ApFY	AA318ApFB	AA323ApFB	AA326ApFB	AA329ApFY	AA333ApFB	AA335ApFB	AA346ApFY	AA348ApFB	AA351ApFY	AA354ApFY	AA355ApFY	AA356ApFY	AA360ApFB	AA364ApFB	AA370ApFY
			appalachiensis	*appalachiensis*	*appalachiensis*	*appalachiensis*	*appalachiensis*	*appalachiensis*	*appalachiensis*	*appalachiensis*	*appalachiensis*	*appalachiensis*	*appalachiensis*	*appalachiensis*	*appalachiensis*	*appalachiensis*	*appalachiensis*	*appalachiensis*	*appalachiensis*	*appalachiensis*	*appalachiensis*	*appalachiensis*	*appalachiensis*	*appalachiensis*	*appalachiensis*	*appalachiensis*	*appalachiensis*	*appalachiensis*	*appalachiensis*	*appalachiensis*	*appalachiensis*	*appalachiensis*	*appalachiensis*
mt gene	COI	18	C	C	C	C	C	C	C	C	C	C	C	C	C	C	C	C	C	C	C	C	C	C	C	C	C	C		C	C		
	COI	93	G	G	G	G	G	G	G	G	G	G	G	G	G	G	G	G	G	G	G	G	G	G	G	G	G	G		G	G		
	COI	393	T	T	T	T	T	T	T	T	T	T	T	T	T	T	T	T	T	T	T	T	T	T	T	T	T	T		T	T		
	COI	402	T	T	T	T	T	T	T	T	T	T	T	T	T	T	T	T	T	T	T	T	T	T	T	T	T	T		T	T		
	COI	489	C	C	C	C	C	C	C	C	C	C	C	C	C	C	C	C	C	C	C	C	C	C	C	C	C	C		C	C		
	COI	558	G	G	G	G	G	G	G	G	G	G	A	G	G	G	A	G	G	G	G	G	G	G	G	G	G	G		G	G		
	COI	627	T	T	T	T	T	T	T	T	T	T	T	T	T	T	T	T	T	T	T	T	T	T	T	T	T	T		T	T		
	COI	642	C	C	C	C	C	C	C	C	C	C	C	C	C	C	C	C	C	C	C	C	C	C	C	C	C	C		C	C		
	COI	687	G	G	G	G	G	G	G	G	G	G	G	G	G	G	G	G	G	G	G	G	G	G	G	G	G	G		G	G		
	COI	768	A	A	A	A	A	A	A	A	A	A	A	A	A	A	A	A	A	A	A	A	A	A	A	A	A	A		A	A		
	COI	789	C	C	C	C	C	C	C	C	C	C	C	C	C	C	C	C	C	C	C	C	C	C	C	C	C	C		C	C		
Z-linked genes	Kettin	72	A	T	T	T		T							T		T		T	T			T		T	T	?	T		T	T		T
	Kettin	78	T	A	A	A		A							A		A		A	A			A		A	A	?	A		A	A		A
	Kettin	148	T	T	C	C		C							T		C		C	C			C		C	C	?	C		C	C		C
	Kettin	157	A	A	A	A		A							A		A		A	A			A		A	A	A	A		A	A		A
	Kettin	215	G	A	A	A		A							A		A		A	A			G		A	A	?	A		A	A		?
	Kettin	401	T	T	A	A		A							T		A		A	A			T		A	A	A	A		A	A		A
	Kettin	683	C	C	A			A							C		A		A	A			C		A	A	A	A		A			A
	Kettin	1040	G	G	A			A							G		A		A	A			G		A	A	A	A		A			
	TH	65	A	G	G	G		G	A	G	G	G	G	G	G				G	G	A	G	A	G	G	G	G	G		G	G	G	
	TH	161	G	C	C	C		C	G	C	C	C	C	C	C				C	C	G	C	G	C	C	C	C	C		C	C	C	
	TH	176	T	G	G	G		G	T	G	G	G	G	G	G				G	G	G	G	G	G	G	G	G	G		G	G	G	
	TH	183	A	G	G	G		G	A	G	G	G	G	G	G				G	G	A	G	A	G	G	G	G	G		G	G	G	
	TH	228	G	A	A	A		A	G	A	A	A	A	A	A				A	A	G	A	G	A	A	A	A	A		A	A	A	
	TH	341	T	T	T	T		T	T	T	T	T	T	T	T				T	T	T	T	T	T	T	T	T	T		T	T	T	
	TH	458	C	C	C	C		C	C	C	C	C	C	C	C				C	C	C	C	C	C	C	C	C	C		C	C	C	
	TH	476	C	T	T	T		T	C	T	T	T	T	T	T				T	T	C	T	T	T	T	T	T	T		T	T	T	
	TH	530	G	A	A	A		A	G	A	A	A	A	A	A				A	A	G	A	G	A	A	A	A	A		A	A	A	
	Tpi	80	T	T	T	T									T		T		T	T		T	T		T	T	T	T		T			T
	Tpi	83	T	T	C	C									T		C		C	C		T	T		T	C	T	C		T			T
	Tpi	106	T	T	T	T									T		T		T	T		–	T		–	T	–	T		T			T
	Tpi	131	T	C	C	C									C		C		C	C		–	T		–	C	–	C		C			C
	Tpi	132	T	C	C	C									C		C		C	C		–	T		–	C	–	C		C			C
	Tpi	134	C	A	A	A									A		A		A	A		–	C		–	A	–	A		A			A
	Tpi	145	T	A	A	A									A		A		A	A		–	T		–	A	–	A		A			A
	Tpi	151	T	G	G	G									G		G		G	G		–	T		–	G	–	G		G			G
	Tpi	153	T	A	A	A									A		A		A	A		–	T		–	A	–	A		A			A
	Tpi	156	G	T	T	T									T		T		T	T		–	G		–	T	–	T		T			T
	Tpi	163	–	T	T	T									T		T		T	T		–	–		–	T	–	T		T			T
	Tpi	171	T	A	–	–									A		–		–	–		–	A		–	–	–	–		–			–
	Tpi	172	A	A	–	–									A		–		–	–		–	A		–	–	–	–		–			–
	Tpi	173	A	A	T	T									A		T		T	T		–	A		–	T	–	T		T			T
	Tpi	194	T	C	C	C									C		C		C	C		–	T		–	C	–	C		C			C
	Tpi	216	T	T	–	–									T		–		T	–		–	T		–	–	–	–		–			–
	Tpi	217	A	A	T	T									A		T		T	T		–	A		–	T	–	T		–			–
	Tpi	219	–	G	T	T									G		T		T	T		T	–		T	T	T	T		–			–
	Tpi	220	–	T	T	T									T		T		T	T		T	–		T	T	T	T		T			T
	Tpi	222	T	T	T	T									T		T		T	T		T	T		T	T	T	T		T			T
	Tpi	231	G	T	T	T									T		T		T	T		T	G		T	T	T	T		T			T
	Tpi	265	C	C	T	T									C		T		T	T		T	C		T	T	T	T		T			T
	Tpi	287	G	G	A	A									G		A		A	A		G	G		G	A	G	A		G			G
	Tpi	318	G	A	A	A									A		A			A		A	G		A	A	A	A		A			A
	Tpi	322	T	A	A	A									A		A			A		A	T		A	A	A	A		A			A
	Tpi	335	C	C	G	G									C		C		?	G		C	C		C	G	C	C		C			C
	Tpi	339	T	T	T	T									T		T		T	T		T	T		T	T	T	T		T			T
	Period	32	G	A	G	C									C		C		C	G		C	G		C	C	C	C	C	C	C		C
	Period	165	G	–	G	–		G							–		–		–	G		–	G		–	–	–	–	–	–	–		–
	Period	172	T	–	T	–		T							–		–		–	T		–	T		–	–	–	–	–	–	–		–
	Period	435	T		T	C									C		C		C	T		C	T		C	C	C	C	C	C	C		C
	Period	465	G		G	A		G							A		A		A	G		A	G		A	A	A	A	A	A	A		A
	Ldh	30	T	T	T	C		T							T				T	T			C		T	T	T	T	C	T			T
	Ldh	96	C	C	C	T		C							C				C	C			C		C	C	C	C	C	C			C
	Ldh	126	T	T	T	A		T							T				T	T			T		T	T	T	T	T	T			T
	Ldh	141	A	G	T	A		G							G				T	A			A		T	T	A	T	A	T			T
	Ldh	142	G	C	C	G		C							C				C	C			G		C	C	C	C	G	C			C
	Ldh	144	A	G	G	A		G							G				G	G			A		G	G	G	G	A	G			G
	Ldh	146	C	C	T	C		C							C				T	C			C		T	T	C	C	C	T			T
	Ldh	207	C	C	C	G		C							C				C	C			C		C	C	C	C	C	C			C
	Ldh	219	C	G	C	C		G							G				C	C			C		C	C	C	C	C	C			C
	Ldh	223	T	T	T	T		T							T				G	T			T		G	G	G	G	T	G			G
	Ldh	237	C	T	C	T		T							T				C	C			C		C	C	C	C	C	C			C
	Ldh	243	A	A	A	–		A							A				A	A			A		A	A	A	A	A	A			A
	Ldh	244	A	C	C	–		C							C				C	C			C		C	C	C	C	C	C			C
	Ldh	262	G	G	G	–		G							G				G	G			G		G	G	G	G	G	G			G
	Ldh	263	T	C	T	–		C							C				T	T			T		T	T	T	T	T	T			T
	Ldh	398	C	C	A	C		C							C				A	C			C		A	A	A	A	C	A			A
	Ldh	416	C			A		C							C				C	C			C		C	C	C	C	C	C			C
	PAH	35	T	C	C	C		T							C		C		C	C		C	C		C	C		C	C	C	C		C
	PAH	102	T	T	T	T		T							C		T		T	C		T	C		T	T		T	T	T	T		T
	PAH	131	C	C	C	C		T							T		C		C	T		C	T		C	C		C	C	C	C		C
	PAH	257	A	G	G	A		A							A		G		G	G		G	G		G	G		G	G	G	G		G
	PAH	286	G	G	A	G		G									G		G	G		A	G		A	A		A	A	A	A		A
	PAH	304	C	A	T	A		C							C		A		A	C		T	A		T	T		T	T	T	T		T
	PAH	310	C	C	A	C		C							C		C		C	C		A	C		A	A		A	A	A	A		A
	PAH	311	C	C	A	C		C							C		C		C	C		A	C		A	A		A	A	A	A		A
	PAH	321	C	C	C	C		–							–		C		C	–		C	C		C	C		C	C	C	C		C
	PAH	327		G	G	G		A							A		G		G	A		G	?		G	G		G	G	G	G		G
	PAH	368	T	C	C	T		T							C		C		C	C		C	T		C	C		C	C	C	C		C
	PAH	470	A	A	A	A		A							A		A		A	C		A	A		A	A		A	A	A	A		A
	PAH	521	–	A	A	A		A							–		A		A	A		A	A		A	A		A	A	A	A		A
	PAH	524	T	A	A	A		A							T		A		A	A		A	A		A	A		A	A	A	A		A
	PAH	526	T	C	C	C		C							T		C		C	C		C	C		C	C		C	C	C	C		C
	PAH	529	C	T	T	C		C							C		T		T	T		T	C		T	T		T	T	T	T		T
	PAH	536	T	A	A	A		A							T		A		A	A		A	A		A	A		A	A	A	A		A
	PAH	541	A	–	–	A		–							A		–		–	–		–	–		–	–		–	–	–	–		–
	PAH	546	G	T	T	G		T							G		T		T	T		T	C		T	T		T	T	T	T		T
	PAH	553	G	S	G	G		C							G		G		C	C		G	C		G	G		G	G	G	G		G
	PAH	559	G	T	T	G		T							G		T		T	T		T	T		T	T		T	T	T	T		T
	PAH	561	C	T	T	C		C							C		T		T	C		T	C		T	T		T	T	T	T		T
	PAH	575	A	A	A	A		C							A		A		A	C		A	C		A	A		A	A	A	A		A
	PAH	578	G	A	A	G		C							G		A		A	C		A	C		A	A		A	A	A	A		A
	PAH	579	G	A	A	G		G							G		A		A	G		A	G		A	A		A	A	A	A		A
	PAH	586	T	C	C	T		C							T		C		C	C		C	C		C	C		C	C	C	C		C
	PAH	588	G	A	A	G		A							G		A		A	A		A	A		A	A		A	A	A	A		A
	PAH	590	C	T	T	C		T							C		T		T	T		T	T		T	T		T	T	T	T		T
	PAH	600	C	T	T	C		C							C		T		T	C		T	C		T	T		T	T	T	T		T
	PAH	638	C	T	T	C		T							?		T		T	C		T	C		T	T		T	T	T	T		T
	PAH	658	T	Y	C	T		T							T		T		C	T		C	T		C	C		C	C	C	C		C
	PAH	677		A	A	G		G							G		A		A	G		A	G			A		A	A		A		A
	PAH	682	A	A	A	A		A							A		A		A	A		A	G		A	A		A	A	A	A		A
	PAH	702	A	G	G			A							A		G		G	A		G	A		G	G		G	G	G	G		G
	PAH	716	T	T	T	C		C							C		T		T	C		T	C		T	T		T	T	T	T		T
	PAH	720	A	T	T	A		A							A		T		T	A		T	A		T	T		T	T	T	T		T
	PAH	738	A	C	T	C		G							A		C		C	A		C	C		C	C		T	C	C	C		C
	PAH	742	–	A	A	–		–							–		A		A	–		A	–		A	A		A	A	A	A		A
	PAH	748	C	T	T	C		C							C		T		T	C		T	C		T	T		T	T	T	T		T
	PAH	753	T	A	A	T		T							T		A		A	T		A	T		A	A		A	A	A	A		A
	PAH	772	C	T		C									C		T		T			T				T			T	T	T		C

Group	Gene	Base	AA456ApM	AA457ApFY	AA458ApFY	AA459ApFY	AA460ApFY	AA461ApFAb	AA462ApFB	AA464ApM	AA465ApM	AA466ApM	AA467ApFY	AA468ApM	AA469ApM	AA470ApM
			appalachiensis	*appalachiensis*	*appalachiensis*	*appalachiensis*	*appalachiensis*	*appalachiensis*	*appalachiensis*	*appalachiensis*	*appalachiensis*	*appalachiensis*	*appalachiensis*	*appalachiensis*	*appalachiensis*	*appalachiensis*
Z-linked genes	TH	65	G	G	G	G	G	G	G	A	?	G	G	G	G	A
	TH	161	C	C	C	C	C	C	C	G	C	C	C	C	C	G
	TH	176	G	G	G	G	G	G	G	T	G	G	G	G	G	G
	TH	183	G	G	G	G	G	G	G	A	G	G	G	G	G	G
	TH	228	A	A	A	A	A	A	A	C	A	A	A	A	A	G
	TH	341	T	T	T	T	T	T	T	T	T	T	T	T	T	C
	TH	458	C	C	C	C	C	C	C	C	C	C	C	C	C	C
	TH	476	T	T	T	T	T	T	T	C	T	T	T	T	T	C
	TH	530	A	A	A	A	A	A	A	A	A	A	A	A	A	A

Group	Gene	Base	AA378CanF	AA379CanF	AA380CanM	AA391CanF	AA392CanF	AA393CanF	AA401CanM	AA403CanM	AA413CanM	AA418CanM	AA419CaF	MA34LFCanM	MA35LFCanM	MA36LFCanM	MA37LFCaM	MA38LFCanM	MA56EFCanF	MA57EFCanF	MA58EFCanF	MA59EFCanM	MA60EFCanF	MA61EFCanM	MA64EFCanM
			canadensis	*canadensis*	*canadensis*	*canadensis*	*canadensis*	*canadensis*	*canadensis*	*canadensis*	*canadensis*	*canadensis*	*canadensis*	*canadensis*	*canadensis*	*canadensis*	*canadensis*	*canadensis*	*canadensis*	*canadensis*	*canadensis*	*canadensis*	*canadensis*	*canadensis*	*canadensis*
mt gene	COI	18	T	T	T	T	T		T	T	T	T	T	T	T	T	T	T	T	T	T	T	T	T	T
	COI	93	G	G	A	G	G		A	A	G	G	A	A	A	A	A	G	G	G	G	G	G	G	G
	COI	393	C	C	C	C	C		C	C	C	C	C	C	C	C	C	C	C	C	C	C	C	C	C
	COI	402	C	C	C	C	C		C	C	C	C	C	C	C	C	C	C	C	C	C	C	C	C	C
	COI	489	T	T	T	T	T		T	T	T	T	T	T	T	T	T	T	T	T	T	T	T	T	T
	COI	558	A	A	A	A	A		A	A	A	G	A	A	A	A	A	A	A	A	A	A	A	A	A
	COI	627	C	C	C	C	C		C	C	C	C	C	C	C	C	C	C	C	C	C	C	C	C	C
	COI	642	T	T	T	T	T		T	T	T	T	T	T	T	T	T	T	T	T	T	T	T	T	T
	COI	687	A	A	A	A	A		A	A	A	A	A	A	A	A	A	A	A	A	A	A	A	A	A
	COI	768	T	T	T	T	T		T	T	T	T	T	T	T	T	T	T	T	T	T	T	T	T	T
	COI	789	T	T	T	T	T		T	T	T	T	T	T	T	T	T	T	T	T	T	T	T	T	T
Z-linked genes	Kettin	72	T	T	T	T	T				T	T	T	A	W	T	T	A	T	T	T	T	T	T	T
	Kettin	78	T	A	A	A	A				A	A	A	W	W	A	A	W	A	A	A	A	A	A	A
	Kettin	148	C	C	C	C	C				T	C	C	T	Y	C	C	Y	C	C	C	C	C	T	T
	Kettin	157	A	T	A	A	A				T	A	A	W	W	W	A	W	T	A	A	A	A	T	T
	Kettin	215	A	A	A	A	A				A	A	A	R	R	A	A	G	A	A	A	A	A	A	G
	Kettin	401	T	A	A	A	A				T	A	A	T	W	A	A	W	A	A	A	A	A	T	T
	Kettin	683	C	A	A	A	A				C	A	A	M	M	A	A	M	A	A	A	A	A	C	C
	Kettin	1040	G	A	A	A	A				G	A	A	R	R	A	A	R	A	A	A	A	A	G	A
	TH	65	G	G	G	G	G	G			G	G	G	?	G	G	G	G	G	G	G	G	G	G	G
	TH	161	C	C	C	C	C	C			C	C	C	C	C	C	C	C	C	C	C	C	C	C	C
	TH	176	G	G	G	G	G	G			G	G	G	G	G	G	G	G	G	G	G	G	G	G	G
	TH	183	G	G	G	G	G	G			G	G	G	G	G	G	G	G	G	G	G	G	G	G	G
	TH	228	A	A	A	A	A	A			A	A	A	A	A	A	A	A	A	A	A	A	A	A	A
	TH	341	T	T	T	T	T	T			T	T	T	T	T	T	T	T	T	T	T	T	T	T	T
	TH	458	C	C	C	C	C	C			C	C	C	C	C	C	C	C	C	C	C	C	C	C	C
	TH	476	T	T	T	T	T	T			T	T	T	T	T	T	T	T	T	T	T	T	T	T	T
	TH	530	A	A	A	A	A	A			A	A	A	A	A	A	A	A	A	A	A	A	A	A	A
	Tpi	80	T	T	T	T	T				T	T		T				T	T	T	T		T	T	T
	Tpi	83	T	C	C	T	C				T	C		G				C	C	T	C		C	T	T
	Tpi	106	T	T	T	T	T				T	T		T				T	T	T	T		T	T	T
	Tpi	131	T	C	C	C	C				C	C		C				C	C	C	C		C	C	T
	Tpi	132	C	C	C	C	C				C	C		C				C	C	C	C		C	C	C
	Tpi	134	G	A	A	G	A				A	A		A				A	A	A	A		A	A	G
	Tpi	145	A	A	A	A	A				A	A		A				A	A	A	A		A	A	A
	Tpi	151	G	G	G	G	G				G	G		G				G	G	G	G		G	G	G
	Tpi	153	A	A	A	A	A				A	A		A				A	A	A	A		A	A	A
	Tpi	156	T	T	T	T	T				T	T		T				T	T	T	T		T	T	T
	Tpi	163	T	T	T	T	T				T	T		T				T	T	T	T		T	T	T
	Tpi	171	C	–	–	–	–				–	–		–				–	–	–	–		–	–	C
	Tpi	172	A	–	–	–	–				–	–		–				–	–	–	–		–	–	A
	Tpi	173	A	T	T	T	T				–	T		T				T	T	T	T		T	–	A
	Tpi	194	C	C	C	C	C				–	C		C				C	C	C	C		C	–	C
	Tpi	216	C	–	–	–	–				T	–		–				–	–	–	–		–	T	C
	Tpi	217	T	T	T	–	T				A	T		–				T	T	–	T		T	A	T
	Tpi	219	C	T	T	T	T				A	T		T				T	T	T	T		T	A	C
	Tpi	220	T	T	T	T	T				T	T		T				T	T	T	T		T	T	T
	Tpi	222	T	T	T	T	T				T	T		T				T	T	T	T		T	T	T
	Tpi	231	T	T	T	T	T				T	T		T				T	T	T	T		T	T	T
	Tpi	265	C	T	T	T	T				C	T		T				T	T	T	T		T	C	C
	Tpi	287	G	A	A	G	A				G	A		R				A	A	G	A		A	G	G
	Tpi	318	C	A	A	A	A				A	A		A				A	A	A	A		A	A	C
	Tpi	322	A	A	A	A	A				G	A		A				A	A	A	A		A	A	A
	Tpi	335	A	G	C	C	G				C	C		C				G	C	C	G		G	C	A
	Tpi	339	T	T	T	T	T				T	T		T				T	T	T	T		T	T	T
	Period	32	C	C	G	C	C				C	C	C	G	C	G	C	C	C	C	C	C	G	C	C
	Period	165	–	–	G	–	–				–	–	–	G	–	G	–	–	–	–	–	–	G	–	–
	Period	172	–	–	T	–	–				–	–	–	T	–	T	–	–	–	–	–	–	T	–	–
	Period	435	C	C	T	C	C				C	C	C	T	C	T	C	C	C	C	C	C	T	C	C
	Period	465	?	A	G	A	A				?	A	?	G	A	G	A	A	A	A	A	A	G	?	A
	Ldh	30		T		T	T				T	T	T	T	T	T	T	T	T	T		T	T	T	T
	Ldh	96		C		C	C				C	C	C	C	C	C	C	C	C	C		C	C	C	C
	Ldh	126		T		T	T				T	T	T	T	T	T	T	T	T	T		T	T	T	T
	Ldh	141		T		T	T				–	A	T	T	T	T	T	T	A	T		A	T	–	A
	Ldh	142		C		C	C				C	C	C	C	C	C	C	C	C	C		C	C	C	G
	Ldh	144		G		G	G				A	G	G	G	G	G	G	G	G	G		G	G	A	A
	Ldh	146		T		C	T				C	C	C	T	T	C	T	T	C	T		C	T	C	C
	Ldh	207		C		C	C				C	C	C	C	C	C	C	C	C	C		C	C	C	C
	Ldh	219		C		C	C				C	C	C	C	C	C	C	C	C	C		C	C	C	C
	Ldh	223		G		G	G				T	T	G	G	T	G	G	K	G	G		T	G	T	T
	Ldh	237		C		C	C				C	C	C	C	C	C	C	C	C	C		C	C	C	C
	Ldh	243		A		A	A				A	A	A	A	A	A	A	A	A	A		A	A	A	A
	Ldh	244		C		C	C				C	C	C	C	C	C	C	C	C	C		C	C	C	C
	Ldh	262		G		G	G				G	G	G	G	G	G	G	G	G	G		G	G	G	G
	Ldh	263		T		T	T				C	T	T	T	T	T	T	T	T	T		T	T	C	T
	Ldh	398		A		?	A				C	A	A	A	A	A	A	A	A	A		A	A	C	C
	Ldh	416		C		?	?				C	?	C	C	C	C	C	C	C	C		C	C	C	C
	PAH	35	C	C	C	C	C				C	C	C	C	C	C	C	C	C	C	C	C	C	C	C
	PAH	102	T	T	T	T	T				T	T	T	T	T	T	T	T	T	T	T	T	T	T	T
	PAH	131	C	C	C	C	C				C	C	C	C	C	C	C	C	C	C	C	C	C	C	C
	PAH	257	G	G	G	G	G				G	G	G	G	G	G	G	G	G	G	G	G	G	G	G
	PAH	286	A	A	A	G	A				A	A	A	A	R	A	A	A	G	A	A	R	A	R	A
	PAH	304	T	T	T	A	T				T	T	T	T	W	T	T	T	A	T	T	W	T	W	T
	PAH	310	A	A	A	C	A				A	A	A	A	M	A	A	A	C	A	A	M	A	M	A
	PAH	311	A	A	A	C	A				A	A	A	A	M	A	A	A	C	A	A	M	A	M	A
	PAH	321	C	C	C	C	C				C	C	C	C	C	C	C	C	C	C	C	C	C	C	C
	PAH	327	G	G	G	G	G				G	G	G	G	G	G	G	G	G	G	G	G	G	G	G
	PAH	368	C	C	C	C	C				C	C	C	C	C	C	C	C	C	C	C	C	C	C	C
	PAH	470	A	A	A	A	A				A	A	A	A	A	A	A	A	A	A	A	A	A	A	A
	PAH	521	A	A	A	A	A				A	A	A	A	A	A	A	A	A	A	A	A	A	A	A
	PAH	524	A	A	A	A	A				A	A	A	A	A	A	A	A	A	A	A	A	A	A	A
	PAH	526	C	C	C	C	C				C	C	C	C	C	C	C	C	C	C	C	C	C	C	C
	PAH	529	T	T	T	T	T				T	T	T	T	T	T	T	T	T	T	T	T	T	T	T
	PAH	536	A	A	A	A	A				A	A	A	A	A	A	A	A	A	A	A	A	A	A	A
	PAH	541	–	–	–	–	–				–	–	–	–	–	–	–	–	–	–	–	–	–	–	–
	PAH	546	T	T	T	T	T				T	T	T	T	T	T	T	T	T	T	T	T	T	T	T
	PAH	553	G	G	G	G	G				G	G	G	G	G	G	G	G	G	G	G	G	G	G	G
	PAH	559	T	T	T	T	T				T/-	–	T	T	T	T	T	T	T	T	T	T/-	T	T	–
	PAH	561	T	T	T	T	T				T	T	T	T	T	T	T	T	T	T	T	T	T	T	T
	PAH	575	A	A	A	A	A				A	A	A	A	A	A	A	A	A	A	A	A	A	A	A
	PAH	578	A	A	A	A	A				A	A	A	A	A	A	A	A	A	A	A	A	A	A	A
	PAH	579	A	A	A	A	A				A	A	A	A	A	A	A	A	A	A	A	A	A	A	A
	PAH	586	C	C	C	C	C				C/?	C	C	C	C	C	C	C	C	C	C	C	C	C	C
	PAH	588	A	A	A	A	A				A/?	A	A	A	A	A	A	A	A	A	A	A	A	A	A
	PAH	590	T	T	T	T	T				T/?	T	T	T	T	T	T	T	T	T	T	T	T	T	T
	PAH	600	T	T	T	T	T				T/?	T	T	T	T	T	T	T	T	T	T	T	T	T	T
	PAH	638	T	T	T	T	T				T/?	T	T	T	T	T	T	T	T	T	T	T	T	T	T
	PAH	658	C	C	C	C	C				C	C	C	C	C	C	C	C	C	C	C	C	C	C	C
	PAH	677	A	A	A	A	A				A/-	A	A	?	A	A	A	A	A	A	A	A	A	A	A
	PAH	682	A	A	A	A	A				A	A	A	A	A	A	A	A	A	A	A	A	A	A	A
	PAH	702	G	G	G	G	G				G	G	G	G	G	G	G	G	G	G	G	G	G	G	G
	PAH	716	T	T	T	T	T				T	T	T	T	T	T	T	T	T	T	T	T	T	T	T
	PAH	720	T	T	T	T	T				T	T	T	T	T	T	T	T	T	T	T	T	T	T	T
	PAH	738	T	C	C	C	T				C	C	C	C	C	C	C	C	C	C	C	C	C	C	C
	PAH	742	A	A	A	A	A				A	A	A	A	A	A	A	A	A	A	A	A	A	A	A
	PAH	748	T	T	T	T	T				T	T	T	T	T	T	T	T	T	T	T	T	T	T	T
	PAH	753	A	A	A	A	A				A	A	A	A	A	A	A	A	A	A	A	A	A	A	A
	PAH	772	T		T	T	T					T	T	T		T	T		T	T	T				T
